# Isolation and Characterization of the Novel Phage JD032 and Global Transcriptomic Response during JD032 Infection of Clostridioides difficile Ribotype 078

**DOI:** 10.1128/mSystems.00017-20

**Published:** 2020-05-05

**Authors:** Tinghua Li, Yan Zhang, Ke Dong, Chih-Jung Kuo, Chong Li, Yong-Qiang Zhu, Jinhong Qin, Qing-Tian Li, Yung-Fu Chang, Xiaokui Guo, Yongzhang Zhu

**Affiliations:** a Department of Microbiology and Immunology/School of Global Health, Chinese Center for Tropical Diseases Research, Shanghai Jiao Tong University School of Medicine, Shanghai, China; b Department of Veterinary Medicine, National Chung Hsing University, Taichung, Taiwan; c Shanghai-MOST Key Laboratory of Health and Disease Genomics, Chinese National Human Genome Center at Shanghai, Shanghai, China; d Department of Laboratory Medicine, Ruijin Hospital, Shanghai Jiao Tong University School of Medicine, Shanghai, China; e Department of Population Medicine and Diagnostic Sciences, College of Veterinary Medicine, Cornell University, Ithaca, New York, USA; f Key Laboratory of Parasite and Vector Biology, Ministry of Health, Chinese Center for Tropical Diseases Research, Shanghai, China; Vanderbilt University

**Keywords:** *Clostridioides difficile*, ribotype 078, bacteriophage, RNA-seq, bacteria-phage interaction, transcriptome

## Abstract

Insights into the interaction between phages and their bacterial hosts are crucial for the development of phage therapy. However, only one study has investigated global gene expression of *Clostridioides* (formerly *Clostridium*) *difficile* carrying prophage, and transcriptional reprogramming during lytic infection has not been studied. Here, we presented the isolation, propagation, and characterization of a newly discovered 35,109-bp phage, JD032, and investigated the global transcriptomes of both JD032 and C. difficile ribotype 078 (RT078) strain TW11 during JD032 infection. Transcriptome sequencing (RNA-seq) revealed the progressive replacement of bacterial host mRNA with phage transcripts. The expressed genes of JD032 were clustered into early, middle, and late temporal categories that were functionally similar. Specifically, a gene (*JD032_orf016*) involved in the lysis-lysogeny decision was identified as an early expression gene. Only 17.7% (668/3,781) of the host genes were differentially expressed, and more genes were downregulated than upregulated. The expression of genes involved in host macromolecular synthesis (DNA/RNA/proteins) was altered by JD032 at the level of transcription. In particular, the expression of the *ropA* operon was downregulated. Most noteworthy is that the gene expression of some antiphage systems, including CRISPR-Cas, restriction-modification, and toxin-antitoxin systems, was suppressed by JD032 during infection. In addition, bacterial sporulation, adhesion, and virulence factor genes were significantly downregulated. This study provides the first description of the interaction between anaerobic spore-forming bacteria and phages during lytic infection and highlights new aspects of C. difficile phage-host interactions.

**IMPORTANCE** C. difficile is one of the most clinically significant intestinal pathogens. Although phages have been shown to effectively control C. difficile infection, the host responses to phage predation have not been fully studied. In this study, we reported the isolation and characterization of a new phage, JD032, and analyzed the global transcriptomic changes in the hypervirulent RT078 C. difficile strain, TW11, during phage JD032 infection. We found that bacterial host mRNA was progressively replaced with phage transcripts, three temporal categories of JD032 gene expression, the extensive interplay between phage-bacterium, antiphage-like responses of the host and phage evasion, and decreased expression of sporulation- and virulence-related genes of the host after phage infection. These findings confirmed the complexity of interactions between C. difficile and phages and suggest that phages undergoing a lytic cycle may also cause different phenotypes in hosts, similar to prophages, which may inspire phage therapy for the control of C. difficile.

## Abstract (precis)

C. difficile is one of the most clinically significant intestinal pathogens. Although phages have been shown to effectively control C. difficile infection, the host responses to phage predation have not been fully studied. In this study, we reported the isolation and characterization of a new phage, JD032, and analyzed the global transcriptomic changes in the hypervirulent RT078 C. difficile strain, TW11, during phage JD032 infection. We found that bacterial host mRNA was progressively replaced with phage transcripts, three temporal categories of JD032 gene expression, the extensive interplay between phage-bacterium, antiphage-like responses of the host and phage evasion, and decreased expression of sporulation- and virulence-related genes of the host after phage infection. These findings confirmed the complexity of interactions between C. difficile and phages and suggest that phages undergoing a lytic cycle may also cause different phenotypes in hosts, similar to prophages, which may inspire phage therapy for the control of C. difficile.

## INTRODUCTION

*Clostridioides* (formerly *Clostridium*) *difficile* is the leading cause of postantibiotic diarrhea, the severity of which ranges from mild diarrhea to severe diarrhea, resulting in pseudomembranous colitis, sepsis, and even death (1). The incidence and mortality rates of Clostridioides difficile infection (CDI) have generally increased in North America and subsequently in European countries over the last couple of decades (2). Since the 2000s, CDI has been predominantly associated with ribotype 027 (RT027) (NAP1/ST1) and, to a lesser extent, ribotype 78 (RT078) (NAP07-08/ST11) (2). Compared to RT027, the lineage RT078 typically affects a younger population (3), is more frequently a community-associated disease agent, and results in higher mortality (4). Recently, the lineage RT078 has been confirmed to be involved in zoonotic transmission (5). Antibiotics, including vancomycin, metronidazole, and fidaxomicin, are currently the most common antibiotics used for the treatment of CDI. However, a median of 21.6% of patients with primary CDI develop recurrent disease within 2 weeks of therapy due to the disruption of native intestinal bacteria by antibiotics (6). In addition, one of the significant risk factors for CDI is the dysbiosis of the normal gut microbiota, which is mainly due to the administration of antibiotics. As such, therapies focusing on the usage of C. difficile phages have been developed as a means to avoid antibiotic-induced clearance of normal intestinal flora (6).

Phages offer several exclusive advantages over antibiotics: they are highly specific to their bacterial hosts, harmless to normal microbiota and humans, able to penetrate the complex biofilms located on the intestinal mucosal surface, and less expensive to produce (7). In fact, since they were discovered in the 1920s, phages have been used widely in Eastern European countries, where they are available as an over-the-counter pharmaceutical product against many bacterial species (8). In addition, with the alarming rise in multiple drug-resistant bacteria, there is an increasing interest in the use of phages as a complement to antibiotic therapy worldwide, and several clinical trials are in process (9).

Currently, 25 complete genomes of C. difficile phages are available in public databases; however, none of these phages are strictly lytic. Several studies have shown that these phages could partially kill C. difficile *in vitro* and *in vivo* (10–13). However, some of them can also lysogenize their C. difficile hosts during the infection process (14). Therefore, it is important to determine whether temperate phages may tend to lysogenize their host bacterium and to evaluate their ability to serve as novel therapeutics against C. difficile infection. For lytic viruses, phage replication relies heavily on host metabolism, and phages must alter the levels of host transcripts for productive infection (15). Hence, it is important to investigate the interaction between the host and the phage.

Studies on phage-host interactions have enhanced our understanding of the evolutionary process of phages, coevolution of phages and hosts, mechanism of phage infection, and effects of phages on host metabolism, as well as the complexity and diversity of phage-bacterium interactions. Therefore, more host-phage systems need to be explored to fully understand phage-host interactions. However, studies focusing on phage-bacterium interactions at the gene expression level have been undertaken mainly in aerobic and facultative anaerobic bacteria, such as Escherichia coli, *Cyanobacterium*, *Bacteroidetes*, Pseudomonas aeruginosa, Lactococcus lactis, Klebsiella pneumoniae, and Mycobacterium smegmatis (16–22). Only one study investigated the effect of a prophage on the transcriptome of C. difficile during lysogeny, which showed that the prophage phiCD38-2 can significantly upregulate the cell wall protein CwpV in C. difficile R20291, therefore conferring phase-variable phage resistance (23). However, the details of phage-induced transcriptional reprogramming in C. difficile during productive infection have not been fully studied.

Here, using the RT078 strain TW11 as a sensitive host, we isolated a phage designated JD032 from the RT078 C. difficile strain TW69 by mitomycin C induction and characterized its morphological and biological properties, *in vitro* bactericidal ability and host spectrum, replication cycle, and genomic information to explore the possibility of using this phage for clinical treatment. On the basis of this, we further investigated the global transcriptome of the interaction between JD032 and its host, the RT078 C. difficile strain TW11, which was isolated from a pig, by a combination of whole-genome sequencing, transcriptome sequencing (RNA-seq), and functional analysis. This analysis was performed to explore the expression pattern of C. difficile phage and unveil the interaction mechanisms between the phage and C. difficile at the transcription level, thereby expanding the understanding of changes in molecular levels after phage-bacterium interaction.

## RESULTS

### Isolation and characterization of phage JD032.

**FIG 1:**
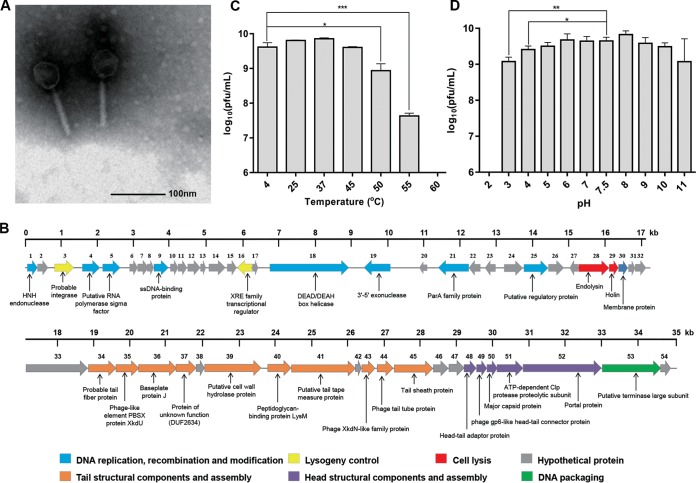
Basic characteristics of phage JD032. (A) TEM of JD032. The diameter of the capsid and the length of the tail were 51.0 ± 1.70 nm and 90.0 ± 2.67 nm, respectively; these measurements were taken on 10 different particles. (B) Genome features of phage JD032. The predicted ORFs and their orientations are represented by arrows. The putative functional assignments are indicated below the ORFs. The functional modules were assigned based on gene annotation and genomic organization and are shown in different colors. (C) Thermostability of phage JD032. The *x* axis shows temperature, and the *y* axis shows the titer of phage JD032 after incubation for 1 h at different temperatures. (D) pH stability of phage JD032. The *x* axis shows pH values, and the *y* axis shows the titers of phage JD032 after incubation for 1 h at different pH values at 37°C. For panels C and D, data are displayed as the means plus standard deviations (SD) (error bars) from three independent experiments. *, 0.01 ≤ *P* < 0.05; **, 0.001 ≤ *P* < 0.01; and ***, *P* < 0.001, respectively.

**FIG 2:**
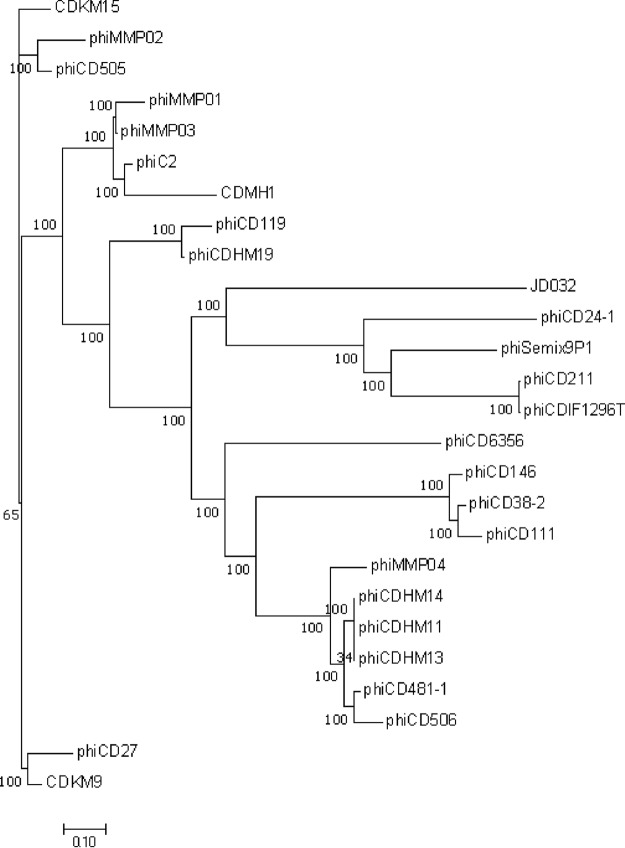
Phylogenetic tree based on the whole genome of the C. difficile phage. The phylogenetic tree was generated using neighbor-joining analysis by MEGA-X. The genome of the C. difficile phage was downloaded from NCBI, and the accession numbers are shown in Table S2 in the supplemental material.

Mitomycin C induction of the C. difficile hypervirulent RT078 strain TW69 yielded a phage, JD032, which formed opaque, small, round plaques of approximately 1.0 mm in diameter on the lawn of another RT078 strain, TW11. This phage could produce plaques on 8/21 of the tested RT078 strains isolated from pigs. Transmission electron microscopy (TEM) analysis of JD032 revealed an icosahedral head with a diameter of 51.0 ± 1.70 nm and a contractile tail with a length of 90.0 ± 2.67 nm (Fig. 1A), suggesting that the phage belongs to the family *Myoviridae* of the order *Caudovirales*. By combination of next-generation sequencing (NGS), the complete genome sequence of JD032 was available and could be further categorized into the phiMMP04 virus-like genus due to its small genome size and the presence of genes encoding both a ParA homolog and Clp protease (24). The genome of JD032 comprised a double-stranded, 35,097-bp circular DNA with an average G+C content of 29.93% and 54 proposed open reading frames (ORFs), 53.7% (29/54) of which had known function (Fig. 1B; see also Table S1 in the supplemental material). It was organized in a typical modular format, including modules for lysogeny control, DNA replication and modification, head structural components and DNA packaging, tail structural components and host cell lysis. In addition, there are no homologs of known virulence or antibiotic resistance genes in the genome of JD032. To determine how JD032 is related to previously reported C. difficile phages, we constructed a phylogenetic tree based on the whole-genome sequence between JD032 and 25 reported C. difficile phages. The resulting tree revealed that JD032 is in an independent clade (Fig. 2 and Table S2), which means a distant evolutionary relationship between JD032 and other C. difficile phages.

10.1128/mSystems.00017-20.3 TABLE S1 Gene annotation and expression data of phage JD032. Download Table S1, PDF file, 0.05 MB. Copyright © 2020 Li et al. 2020 Li et al. This content is distributed under the terms of the Creative Commons Attribution 4.0 International license.

10.1128/mSystems.00017-20.4 TABLE S2 General features and accession numbers of phages used in this study. Download Table S2, PDF file, 0.2 MB. Copyright © 2020 Li et al. 2020 Li et al. This content is distributed under the terms of the Creative Commons Attribution 4.0 International license.

The infectivity of phage JD032 was stable between 4 and 45°C but completely lost at temperatures above 60°C. When incubated at 50°C and 55°C, the titer decreased by 0.68 and 2 log units, respectively, compared with that at 4°C (Fig. 1C). In addition, phage JD032 was stable between pH 5.0 and pH 11.0 (Fig. 1D) and decreased by only 0.57 and 0.24 log units at pH 3.0 and pH 4.0, respectively, compared with pH 7.5. Overall, phage JD032 virions were tolerant to wide pH ranges and high temperatures, which is similar to other C. difficile phages, such as ФHN10, ФHN16-1, ФHN16-2, and ФHN50 (25).

**FIG 3:**
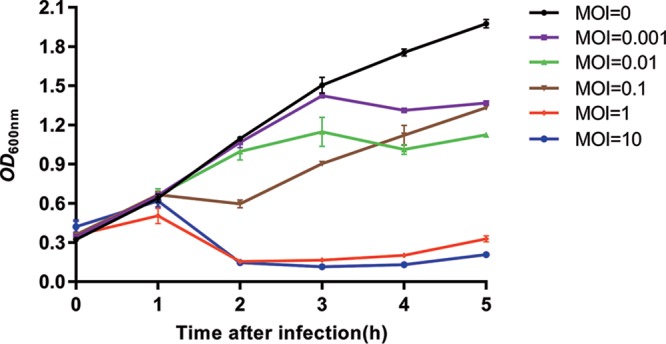
*In vitro* bactericidal activity of phage JD032 against C. difficile strain TW11. C. difficile strain TW11 was infected by phage JD032 at MOIs of 0.001, 0.01, 0.1, 1, and 10 and cultured for up to 5 h. Data are displayed as the means ± SD (error bars) from three independent experiments.

To identify the *in vitro* bactericidal activity of phage JD032, C. difficile strain TW11 was cultured to early log phase and then infected with phage JD032 at a series of multiplicity of infection (MOI) values at 37°C in anaerobic conditions. As shown in Fig. 3, the great majority of TW11 cells were killed by phage JD032 within 2 h of infection when the MOI was equal to or greater than 1. This indicates that JD032 has a very strong bactericidal ability and can effectively control the levels of C. difficile *in vitro*.

### Infection profile of phage JD032.

**FIG 4:**
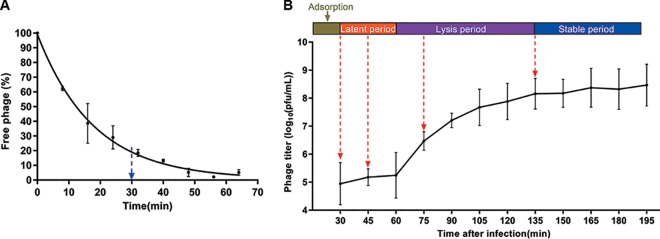
Lytic cycle of phage JD032 against C. difficile strain TW11. (A) Adsorption curve of JD032 to its host C. difficile TW11. The *x* axis shows the incubation time of JD032 and its host, and the *y* axis shows the percentage of the phage that did not adsorb to the host. (B) One-step growth curve of phage JD032. The *x* axis shows the incubation time of JD032 with its hosts after absorption for 30 min; the *y* axis shows the phage titers in the mixture at different times. Data are displayed as the means ± SD from three independent experiments.

The adsorption curve and one-step growth curve of JD032 were examined. As shown in Fig. 4A, more than 80% of JD032 adsorbed to strain TW11 within 30 min, and the ultimate adsorption rate was as high as 98%. As shown in Fig. 4B, the latent period of phage JD032 was ca. 30 min and is therefore similar to other reported latent periods for C. difficile phages (commonly ranging from 32 to 118 min [26, 27]). The number of phage particles reached a peak at 135 min after phage infection, and the burst size was ca. 175 PFU per infected cell, more than other C. difficile phages, which ranged from 5 to 122 phages/cell (26, 28).

### General transcriptomic dynamics of phage JD032 infection.

Based on the replication cycle of JD032 (Fig. 4), four time points (30, 45, 75, and 135 min) that span the entire replication cycle of the phage were selected for exploring the transcriptomic profiles of both JD032 and host C. difficile strain TW11 by RNA-seq, with the phage-uninfected host cells at 0 min as a control. Each experiment was repeated three times.

**FIG 5:**
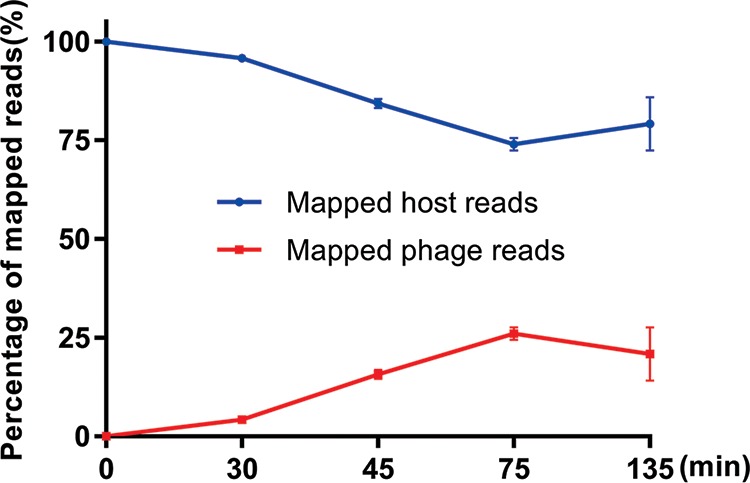
Alignment of RNA read sets against the C. difficile (blue) or phage JD032 (red) genome at different time points after infection. Data are displayed as the means ± SD from three independent experiments.

RNA-seq analysis of the global transcriptome of phage JD032 and strain TW11 over a single phage infection cycle revealed that host mRNA was progressively replaced by phage transcripts, starting from 4.2% at 30 min and peaking at 26.01% at 75 min (Fig. 5). This represents a relative accumulation of phage transcripts.

### Temporal expression patterns of phage JD032.

**FIG 6:**
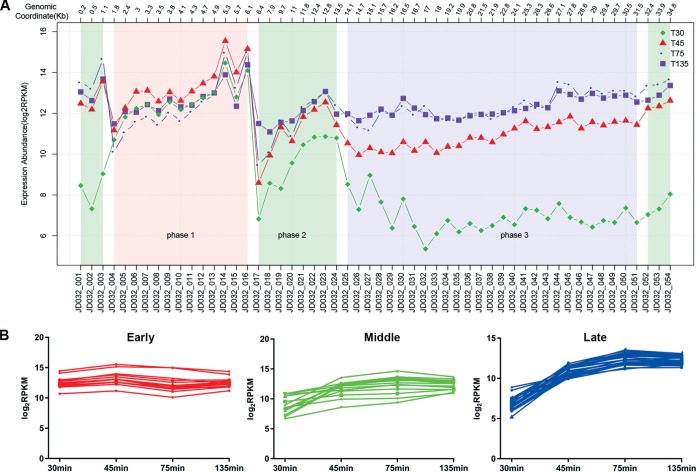
Temporal kinetic transcriptional profile of phage JD032. (A) According to the expression abundance of phage genes at various time points, we divided these into three expression patterns. Genes that are highly expressed at 30 min (T30), 45 min (T45), and 75 min (T75) are called early, middle, and late genes, respectively. (B) Graphs displayed below the subclasses show expression profiles of the individual genes in that subclass as a function of time after infection.

According to the order when the transcript abundances peaked (Fig. 6A and Table S1) (29), 54 phage genes were clustered into three temporal categories. The expression curves are color coded based on this classification (Fig. 6B), where the genes in the expression peak appearing first during infection were designated early genes (red), the genes in the second peak were designated middle genes (green), and the genes in the third peak were designated late genes (blue). Genes in each temporal category were arranged adjacent to each other on the genome and are therefore functionally similar.

The early genes (*JD032*_*orf004*-*016*) were related to host takeover, which initiates phage gene expression and alters the functions of host proteins, including two putative RNA polymerase sigma factors (JD032_ORF004 and JD032_ORF005) and a single-stranded DNA-binding protein (JD032_ORF009). In addition, as a temperate phage, the early genes of JD032 may also be involved in determining the phage replication cycle, since JD032_ORF016, the XRE family transcriptional regulator, is similar to the repressor proteins CI of phage lambda and xenobiotic response element (XRE) of the Bacillus subtilis defective prophage PBSX, which are both involved in the lysogenic-lytic decision.

The middle-expressed genes (*JD032*_*orf001*-*003*, *-017*-*024*, and -*052*-*054*) were DNA metabolism genes involved in DNA replication, recombination/repair, and inheritance. These genes included a DEAD/DEAH box helicase (JD032_ORF018), 3′-5′ exonuclease (JD032_ORF019), ParA family protein (JD032_ORF021), His-Asn-His (HNH) endonuclease (JD032_ORF001), probable integrase/recombinase YoeC OS (JD032_ ORF003), and phage-encoded terminase required for DNA packaging.

Genes expressed late were the structural and lysis genes, including head structural components and DNA packaging (JD032_ORF037, -044, and -048-051), tail structural components (JD032_ORF034-36, -039-041, and -045), holin (JD032_ORF029), and endolysin (JD032_ORF028).

### Complete genome sequence of C. difficile RT078 strain TW11.

The complete genome of strain TW11 was sequenced for subsequent analysis of host-phage interactions using a combination of long-read (PacBio) and short-read (Illumina) sequencing. A single circularized chromosome and a plasmid were generated. The chromosome of C. difficile strain TW11 has a size of 4,100,340 bp (29% G+C) and contains 3,734 predicted coding sequences (CDSs), 35 rRNAs, 89 tRNAs, and 302 other noncoding RNA genes. The plasmid has a size of 42,254 bp (25% G+C) and contains 47 CDSs. In addition, strain TW11 is predicted to encode 7 CRISPR-Cas arrays (Table S3), 8 restriction-modification (RM) system genes (Table S4), 7 pairs of toxin-antitoxin (TA) systems (Table S5), 3 incomplete prophages, and 13 genomic islands. It is worth mentioning that although JD032 is induced from C. difficile RT078 strain TW69, its host strain TW11 does not contain a JD032-like phage sequence, thus, the alignment of the RNA-seq reads to the genome of phage JD032 or strain TW11 will not be confused.

10.1128/mSystems.00017-20.5 TABLE S3 Predicted CRISPR-Cas systems of C. difficile TW11. Download Table S3, PDF file, 0.3 MB. Copyright © 2020 Li et al. 2020 Li et al. This content is distributed under the terms of the Creative Commons Attribution 4.0 International license.

10.1128/mSystems.00017-20.6 TABLE S4 Predicted restriction-modification (RM) system genes and their transcript levels at four time points during phage JD032 infection. Download Table S4, PDF file, 0.2 MB. Copyright © 2020 Li et al. 2020 Li et al. This content is distributed under the terms of the Creative Commons Attribution 4.0 International license.

10.1128/mSystems.00017-20.7 TABLE S5 Predicted toxin-antitoxin (TA) system genes and their transcript levels at four time points during phage JD032 infection. Download Table S5, PDF file, 0.2 MB. Copyright © 2020 Li et al. 2020 Li et al. This content is distributed under the terms of the Creative Commons Attribution 4.0 International license.

### Host responses of C. difficile strain TW11 to phage JD032 infection.

**FIG 7:**
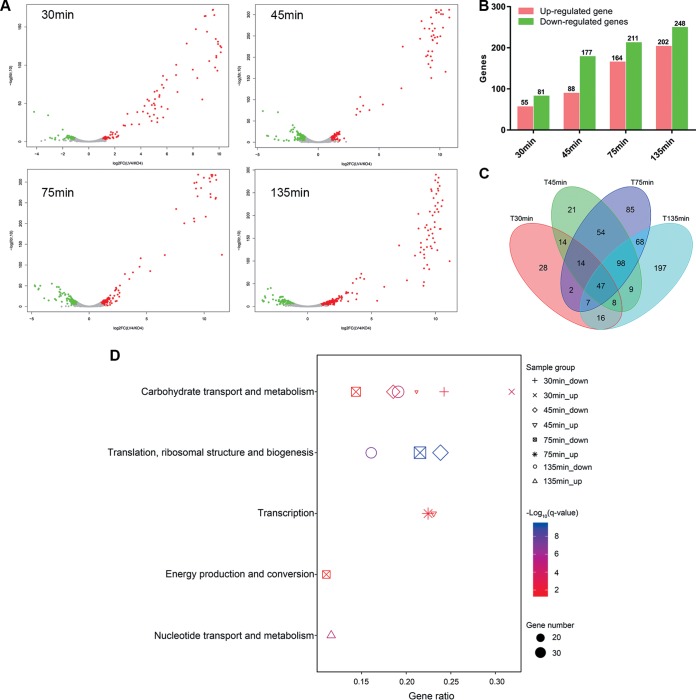
Impact of phage JD032 infection on its host transcriptome. (A) Volcano plot of the C. difficile transcriptome following phage infection compared with the uninfected control. Each dot represents an open reading frame, with upregulated genes shown in red and downregulated genes in green. (B) Number and distribution of DEGs at different infection stages. (C) The Venn diagram shows the intersection of the number of DEGs at each time point. (D) Significant enrichment COG categories of host DEGs (up- and downregulated genes) at each time point after JD032 infection. The shape of the point indicates the time points. The enrichment q-value of each pathway was normalized as negative log_10_ *P* value and is shown as a color gradient. The number of genes enriched in each pathway is represented by the size of the points.

To investigate host responses against phage infection, we analyzed the differentially expressed genes (DEGs) and pathways in C. difficile during infection at an MOI of approximately 10. Globally, comparing the infected samples to the uninfected samples, 17.7% (668/3781) C. difficile genes were differentially expressed. The number of DEGs increased over time in response to phage infection, with more DEGs downregulated than upregulated at all time points (Fig. 7A and B). It is worth mentioning that the number of DEGs per time point did not add up to the total number of DEGs (668, not 1,226) because some genes showed diverse expression patterns at different time points (Fig. 7C). In addition, 11 genes fluctuated between up- and downregulation.

### (i) Functional analysis of host DEGs.

To gain a broader view of the functions of the DEGs, COG (cluster of orthologous group) functional annotation of the upregulated and downregulated DEGs at each time point was performed. The DEGs had a significant functional enrichment among the four time points (Fig. 7D). In the early stage of infection, the DEGs were mainly involved in carbon transport and metabolism (30 min). As the infection progressed, the functional categories of the DEGs changed. Generally, the upregulated genes were mainly involved in transcription (45 and 75 min) and nucleic acid transport and metabolism (135 min), while the downregulated genes were mainly related to translation, ribosomal structure and biogenesis (45, 75 and 135 min), and energy production and conversion (75 min) (Fig. 7D).

In addition, 29.49% (197/668) of the DEGs were enriched in 17 KEGG (Kyoto Encyclopedia of Genes and Genomes) pathways. Based on the adjusted *P* value (*q*) and gene number (GN) of each KEGG pathway, only five pathways were significantly changed (*q* < 0.05 and GN > 3) at one time point or more. The pathway categories of upregulated and downregulated DEGs were markedly different; the pathways carbohydrate metabolism (0 to 75 min), membrane transport (0 to 75 min) and nucleotide metabolism (135 min) were upregulated, but the pathways translation (45 to 135 min), energy metabolism (75 min), and carbohydrate metabolism (135 min) were downregulated (Table S7).

### (ii) Host metabolism was reprogrammed by phage JD032.

Not surprisingly, given what is known about other viral replication pathways, such as those for T4, lambda, mycobacteriophage SWU1, and myovirus NCTC 12673 (22, 30), we identified several genes involved in DNA synthesis, repair and recombination, transcription and translation, as well as amino acid and nucleotide metabolism differentially expressed upon phage JD032 infection of C. difficile.

In terms of genes associated with DNA replication and repair, type I DNA topoisomerase (*TW11_2454* and *TW11_3730*), transposase (*TW11_2823* and *TW11_3409*), and helicase (*TW11_2886*) were downregulated for at least one time point (Table S6).

10.1128/mSystems.00017-20.8 TABLE S6 DEGs in C. difficile strain TW11 upon phage JD032 infection. Download Table S6, PDF file, 0.6 MB. Copyright © 2020 Li et al. 2020 Li et al. This content is distributed under the terms of the Creative Commons Attribution 4.0 International license.

10.1128/mSystems.00017-20.9 TABLE S7 Significantly enriched KEGG pathways of DEGs in C. difficile strain TW11. Download Table S7, PDF file, 0.01 MB. Copyright © 2020 Li et al. 2020 Li et al. This content is distributed under the terms of the Creative Commons Attribution 4.0 International license.

For transcription-related genes, the expression of RNA polymerase subunit β (RpoB, TW11_03589), β’ (RpoC, TW11_3588), and ω (RpoZ, TW11_1121), as well as the major sigma factor RpoD (TW11_2270 and TW11_2227), were not changed at any time points, but the subunit α RpoA (TW11_3553) was significantly downregulated from 45 min to 135 min. It is worth mentioning that genes that make up the *ropA* operon, including ribosomal protein L17 (TW11_3552), S4 (TW11_3554), S11 (TW11_3555), S13 (TW11_3556), L36 (TW11_3557), and initiation factor IF-1 (TW11_3558), were all significantly downregulated after 45 min. As transcriptional regulators play central roles in the control of gene transcription by RNA polymerase, we next analyzed the expression of transcriptional regulators and found that 20.5% (57/237) of the predicted transcriptional regulators were differentially expressed upon JD032 infection; among them, 71.9% were upregulated (Table S6). The altered expression of many transcriptional factors suggests extensive transcriptional reprogramming in response to JD032 infection and multiple possible impacts on bacterial host metabolism.

In addition, many genes involved in translation, ribosomal structure and biogenesis-related genes were significantly downregulated, including genes linked to aminoacyl-tRNA biosynthesis, ribosome, translation factors, tRNA biogenesis, and mRNA biogenesis (Table S6).

The above results indicate that phage infection affects every process of the host macromolecular synthesis (DNA/RNA/proteins) at the level of transcription, which may suggest that JD032 exploits the metabolic mechanisms of the host by various ways to preferentially accomplish its replication.

A total of 106 genes in C. difficile strain TW11 were annotated as related to purine and pyrimidine metabolism; 1 gene was upregulated, and 8 genes were downregulated during the latency of phage infection. During the lysis phase and stable phase, 3 and 17 genes, respectively, were upregulated, and 6 and 10 genes, respectively, were downregulated. Overall, at 30 min, 45 min, and 75 min, downregulation was much more pronounced than upregulation with respect to gene activity. Conversely, when infection stabilized, the upregulated genes were expressed at higher levels than the downregulated genes. This change is opposite what has been observed with M. smegmatis and Campylobacter jejuni after infection with the lytic bacteriophages SWU1 (30) and NCTC 12673 (22), respectively, but similar to P. aeruginosa after infection with the lysogenic phage PaP3 (31).

### (iii) Gene expression of antiphage systems was altered in response to JD032 infection.

The genome of strain TW11 is predicted to encode seven CRISPR-Cas arrays (Table S3), nine RM system genes (Table S4), and seven pairs of TA system genes (Table S5). Of these genes, eight genes (*TW11_0693-0700*) belonging to CRISPR-Cas type I-B were downregulated at least at one time point upon phage infection (Table S6), one RM gene (*TW11_2454*) was downregulated at 75 min postinfection (Table S4), and 43% TA genes were differentially expressed with more upregulated genes than downregulated genes (Table S5). In addition, four genes (*TW11_0457*, *TW11_1619*, *TW11_1706*, and *TW11_1954*) involved in the prokaryotic defense system were downregulated at least at one time point upon phage infection. These results suggest that the host defense system may play a role in the process of phage JD032 infection, but at the same time, JD032 also inhibits expression of some of the defense genes of the host through some currently unknown mechanism, thereby finally achieving effective infection.

### (iv) Effects of JD032 infection on host pathogenicity.

Previous studies suggest that phages present in a lysogenic state (prophage) can influence the overall fitness and virulence of C. difficile (23, 27, 32–34); for example, the expression of the pathogenicity locus (PaLoc) genes *tcdA*, *tcdB*, *tcdR*, *tcdE*, and *tcdC* were downregulated in phiCD119 lysogens (33), and the expression of the cell surface protein gene *cwpV* was upregulated in phiCD38-2 lysogens. However, it is not known whether the phage in the lytic cycle affects the expression of such genes. Therefore, the expression of genes related to virulence and fitness was analyzed.

We found that the expression levels of four cell surface protein genes, *cwp2* (*TW11_0826*), *cwp9* (*TW11_0821*), *cwp26* (*TW11_0956*), and *cwp28* (*TW11_2806*), were changed during phage infection (Table S6). Among these genes, *cwp2* plays a role in toxin A production and bacterial adhesion (35). In addition, two virulence-related genes (*TW11_0828* and *TW11_2172*) were downregulated. *TW11_0828* encodes the cell wall-binding repeat-containing protein Cwp66, which has been confirmed to be an adhesin (36, 37), and *TW11_2172* encodes a hemolysin. Furthermore, the RNA polymerase sporulation sigma factor *sigH* (*TW11_3599*) was downregulated at 45 min, 75 min, and 135 min after JD032 infection. SigH has been proven to be a key sigma factor in the transition phase that controls sporulation, metabolism, and virulence factor expression in C. difficile at the onset of the stationary phase, and the *sigH* mutant is unable to sporulate (38). Since sporulation and adhesion are important factors in the spread and pathogenicity of C. difficile, these results indicate that phage JD032 not only directly kills most bacteria but also may reduce the pathogenicity of the surviving pathogens.

### Verification of phage genes and DEGs by RT-qPCR.

**FIG 8:**
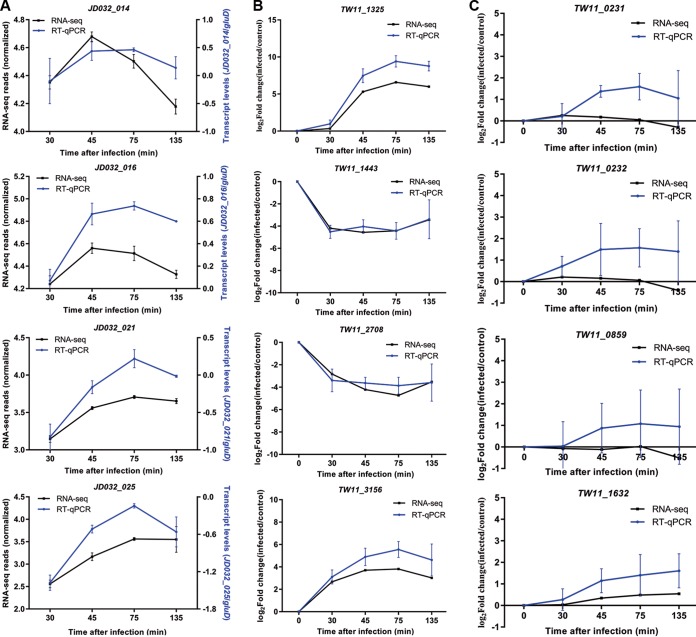
RT-qPCR verification of RNA-seq results. (A) Comparison of the expression levels of four phage genes measured by RT-qPCR and RNA-seq. RT-qPCR data were normalized using glutamate dehydrogenase (gluD) as an internal reference, and the relative expression level was calculated using the 2^-ΔCт^ method. RNA-seq data were normalized to gene length and library size (RPKM). The replicates were averaged and presented as log_10_. (B andC) Verification using RT-qPCR for four DEGs (B), two TA system genes (C), one RM system gene (C), and one unaffected gene (C) of C. difficile strain TW11 upon infection. The relative expression levels of RT-qPCR data and RNA-seq were calculated using the 2^-ΔΔCт^ method and fold change, respectively. The replicates were averaged and are presented as log_10_.

To confirm the reliability of the RNA-seq data, four phage genes (*JD032*_*orf014*, *-orf016*, -*orf021*, and -*orf025*) and four bacterial DEGs (*TW11_1325*, *TW11_1443*, *TW11_2708*, and *TW11_3516*) were randomly selected for reverse transcription-quantitative PCR (RT-qPCR) verification. The RT-qPCR results showed a similar expression trend of the four phage genes compared to RNA-seq (Fig. 8A). For bacterial DEGs, although the fold change of *TW11_1325* and *TW11_3516* was higher with RT-qPCR than with RNA-seq, the pattern of change was similar (Fig. 8B). In addition, we verified the expression of two TA system genes (*TW11_0231* and *TW11_0232*), one RM system gene (*TW11_0859*) and one unaffected host gene (*TW11_1632*). Consistent with RNA-seq, RT-qPCR results also showed that the expression of these genes did not change during phage infection (Fig. 8C).

## DISCUSSION

To gain insight into the interaction between C. difficile and phage under lytic infection, we first reported the isolation and characterization of a new C. difficile phage, JD032, and then characterized the global transcriptional responses between JD032 and C. difficile strain TW11.

Similar to other phages (20, 21, 29, 31, 39–43), the gene expression patterns of phage JD032 also demonstrate three temporal expression classes of early, middle, and late genes and suggest that the transcription patterns of phages may be independent of bacterial host type, phage type, infection efficiency, and environment. However, unlike other temperate phages that have been reported (44, 45), the integrase gene of JD032 (*JD032_orf003*) is highly expressed after the 45-min time point. Integrase is often considered a gene marker of temperate phages at present and is either not expressed or underexpressed during phage infection (22, 46). One study has shown that the concentration of integrase determines the life cycle of mycobacteriophages (47), with high concentrations promoting the establishment of a lysogenic state. Hence, we speculate that JD032 has a strong tendency to integrate into TW11 in the process of infection. This may be one of the reasons why it is difficult to screen for lytic phages that target C. difficile because C. difficile phages may be more inclined to enter the lysogenic cycle.

Early studies on bacterium-phage interactions suggested that phage infection led to a complete shutdown of host transcription (48, 49). However, in the last decade, studies using transcriptome analysis to assess bacterial response to phage infection have shown that this is not the case, with a range of responses in different bacterium-phage systems (16, 19, 22, 29, 39, 41, 50, 51). Viral infections do share common traits. An immediate transient response followed by a subsequent response was found to be common across most viral replicative systems. However, our study also shows a subsequent decrease in transcription levels, which may be related to the time point of infection we studied. As our study covered all stages of viral replication, while most studies did not investigate changes in host gene expression at the stable phase of phage infection. Here, in the stable phase (135 min), the JD032 phage and the TW11 host are in a relatively balanced state wherein most TW11 cells were lysed (see Text S1 and Fig. S1 in the supplemental material), while the remainder were uninfected host or in a lysogenic state (31). Our results indicated that the response of strain TW11 to phage JD032 during the stable phase was significantly different from the response in other periods. For example, as shown in Fig. 7C, there were more bacterial DEGs at the 135-min time point compared with those during other periods. In addition, the significantly enriched KEGG pathways of the DEGs were also significantly different from the other three periods (Table S7). Furthermore, most bacteria experience a massive change in host gene expression upon phage infection. The trend of expression changes varies depending on the host-phage system. Our study indicates that C. difficile phage infection downregulates far more host genes than it upregulates.

10.1128/mSystems.00017-20.1 TEXT S1 Viability of C. difficile TW11 at 135 min after phage JD032 infection. Download Text S1, DOCX file, 0.02 MB. Copyright © 2020 Li et al. 2020 Li et al. This content is distributed under the terms of the Creative Commons Attribution 4.0 International license.

10.1128/mSystems.00017-20.2 FIG S1 Number of viable C. difficile TW11 cells corresponding to OD_600_ values. Download FIG S1, TIF file, 1.9 MB. Copyright © 2020 Li et al. 2020 Li et al. This content is distributed under the terms of the Creative Commons Attribution 4.0 International license.

We found that some antiphage genes in strain TW11 were differentially expressed upon JD032 infection. Recent advances in phage-host interactions have revealed that bacteria have an impressive arsenal of defense mechanisms to proliferate in phage-rich environments, and in response, phages have evolved counterstrategies to evade these antiviral systems (52). More recently, studies have shown that the ability of broad-spectrum host phages to infect multiple hosts is more likely to depend on the effectiveness of host defense strategies than on the differential tailoring of the phage infection process (39), and inhibition of the host defense system is one of the important factors for effective phage infection (21, 29). The upregulation of some TA system genes may suggest the antiphage responses of host TW11 to prevent JD032 infection, while the downregulation of some CRISPR-associated proteins and RM system genes may indicate that JD032 inhibits the host defense system to ensure its effective infection.

During the process of phage infection, a few bacteria may mutate and become resistant to phage infection, which is one of the problems currently faced by phage therapy (53, 54). Our study found that during the lytic infection cycle, some genes related to sporulation and virulence are downregulated, which may suggest that although a few bacteria survive phage infection, their pathogenicity is affected, which brings new hope for supporting phage therapy.

There are unavoidable flaws in this study. First, although a high MOI (MOI = 10) was selected to ensure that more bacteria were infected by phage, the proportion of infected bacteria is unknown. A single phage-bacterium system may solve this problem. In addition, because this study investigated only the transcriptional levels of interaction between the phage and host, some descriptions and discussions need further research to confirm.

In conclusion, we isolated and characterized a new C. difficile phage, JD032, and further provided a general description of the global transcriptional interaction between this phage and its host TW11. We found reprogramming of the host metabolism by phage, antiphage-like responses of the host, repression of the antiphage system by phage, and suppression of pathogenicity-related genes of the host after phage infection at the transcriptome level. This study enhances our knowledge relevant to phage-bacterium interactions, and further research that validates some of these descriptions will be of great significance.

## MATERIALS AND METHODS

### Bacterial strains and culture conditions.

Eighteen Clostridioides difficile RT078 strains were used in this study, all of which were isolated from pigs and kindly donated by Chih-Jung Kuo (Department of Veterinary Medicine, National Chung Hsing University, Taiwan). All strains were routinely grown on prereduced brain heart infusion (BHI) (Oxoid) agar or tryptose-yeast extract (TY) (3% tryptose and 2% yeast extract) broth at 37°C under an anaerobic atmosphere (10% H_2_, 10% CO_2_, and 80% N_2_) inside an anaerobic chamber.

### Induction, propagation, and purification of phage JD032.

Phage induction was performed as previously described by Fortier and Moineau (55) with a few modifications. Details were as follows. Strain TW69 was inoculated into prereduced TY broth and grown at 37°C under anaerobic conditions until the stationary phase (12 to 14 h). A 3% inoculum from an overnight culture was transferred into fresh prereduced TY broth and grown until the early logarithmic phase (optical density at 600 nm [OD_600_] of 0.3 to 0.5). Mitomycin C was added to a final concentration of 3 μg/ml. Cultures containing mitomycin C were further cultured at 37°C under anaerobic conditions for 8 h. Crude lysates were then centrifuged and filtered (0.45-μm-pore-size filter) for phage isolation, and the sensitive host of each phage was determined on the basis of plaque formation.

To further isolate a single strain of phage, phage isolation was conducted by using the double-layer agar method (56). The isolated single phage was named JD032. JD032 was further enriched by using polyethylene glycol 8000 (PEG 8000) and purified by CsCl density gradient ultracentrifugation and dialysis (10 kDa). The purified phage, JD032, was stored in SM buffer at 4°C until further analysis.

### Host range determination.

The host range activity of phage JD032 was determined by spot tests. Details were as follows. Aliquots (400 μl) of the logarithmic-phase cultures (OD_600_ = 0.4 to 0.6) of each tested strain were collected to make double-layer agar plates. Six microliters of the phage stock solution was dropped onto the double-layer plates and allowed to stand at room temperature until absorbed. The plates were then incubated at 37°C in anaerobic conditions overnight and examined for the presence or absence of plaques.

### Morphological characterization.

The morphology of the JD032 phage was negatively stained by phosphotungstic acid and observed by transmission electron microscopy (TEM). Briefly, 400-μl samples of the purified phage particles were washed twice with 0.1× phosphate-buffered saline (PBS) and then fixed with 2% glutaraldehyde for 30 min. After centrifugation (40,000 × *g*, 4°C, 1 h), the pellets were suspended in 0.1× PBS (50 to 100 μl). A 10-μl aliquot of fixed phage particles was spotted onto a carbon-coated copper grid and allowed to absorb for 3 to 5 min. The sample was then negatively stained with 2% (wt/vol) potassium phosphotungstate (pH 7.0) for 2 to 3 min before phage morphology was observed using a transmission electron microscope.

### Biological characteristics and bacteriolytic activity *in vitro*.

The thermostability of phage JD032 was tested using 10^8^ PFU/ml purified phage lysate that was subjected to different temperatures (4, 25, 37, 45, 50, 55, 60, and 65°C) for 1 h. To test pH stability, the purified phage lysate was incubated at different pH ranges from pH 2.0 to pH 11.0 for 1-h intervals. After incubation in a temperature-controlled water bath or pH-controlled environment, the phage activity was determined by a double-layer plating experiment. The results are represented as phage titers at different temperatures or pH ranges. All experiments were repeated in triplicate.

To determine the bacteriolytic activity of phage JD032, C. difficile strain TW11 cultured in TYCM broth (TY broth containing 5 mM CaCl_2_ and MgCl_2_) was infected with phage JD032 at a multiplicity of infection (MOI) of 0.001, 0.01, 0.1, 1, or 10 at 37°C under anaerobic conditions. Culture samples were collected at 60-min intervals for 8 h, and bacterial growth was measured based on the OD_600_. All experiments were repeated in triplicate.

### Adsorption curve and one-step growth curve.

Phage absorption experiments were performed according to the method of Cui et al. (57) with some modifications. Briefly, 5 ml of strain TW11 was grown in TYCM broth to a density of 10^7^ CFU/ml in the early logarithmic stage. Cells were then infected with purified phage JD032 at an MOI of 0.01. The mixture was incubated anaerobically at 37°C. Free phages were sampled immediately after phage addition (0 min) and every 8 min for 64 min by centrifuging the cells for 30 s at 16,000 × *g*, and the phage titer was measured by plating serial dilutions.

One-step growth curves were performed by the method of Zhao et al. (31) with slight modifications; 5 ml of exponentially growing cells was infected with purified JD032 at an MOI of 0.01 and centrifuged for 30 s at 16,000 × g after a 30-min adsorption (by 30 min, 80% of phage JD032 had adsorbed to host TW11 (Fig. 4A). The pellets were washed twice with prereduced TYCM broth to remove unadsorbed phages and then resuspended in 5 ml of fresh prereduced TYCM (30 min). The phage concentration was sampled immediately after resuspension (30 min) and every 15 min for 3 h by centrifugation for 30 s at 16,000 × *g*, and the phage titer was measured by plating a serial dilution of the suspension. The burst time and burst size were calculated based on a one-step growth curve.

### Genome sequencing of phage JD032.

Whole-genome sequencing of JD032 particles was performed using the Illumina MiSeq system at Shanghai Personal Biotechnology Co., Ltd. A total of 3,214,720 high-quality reads were acquired. The sequence assembly and correction were conducted by using SPAdes v3.9.0 (58) and pilon v1.18 (59), respectively. Possible open reading frames (ORFs) were predicted by Glimmer 3.02 (60). All ORFs were annotated using the NR and Swiss-Prot databases. Virulence factors and resistance genes were predicted with the virulence factor database (VFDB) (http://www.mgc.ac.cn/VFs/main.htm) and antibiotic resistance gene database (ARDB) (http://ardb.cbcb.umd.edu/), respectively. The genome sequence of phage JD032 was deposited in GenBank under accession number MK473382.

### Genome sequencing of C. difficile strain TW11.

The genomic DNA of C. difficile strain TW11 was extracted by Puregene Yeast/Bact. kit B (Qiagen) according to the manufacturer’s instructions. Whole-genome sequencing was performed at Shanghai Personal Biotechnology Co., Ltd., using the PacBio RS II and Illumina MiSeq platforms. The *de novo* assembly of the whole-genome sequence was verified by a combination of A5-MiSeq v20150522, CANU, pilon, and mummer software. Gene prediction and annotation were conducted by Glimmer 3.02 (60) and BLAST plus (61), respectively, using the Refseq database.

### RNA-seq library preparation and sequencing.

Samples were collected (10 ml) at 0, 30, 45, 75 and 135 min after infection with JD032 at an MOI of 10 and centrifuged at 16,000 × *g* for 5 min. The supernatant was then removed, and the pellets were washed with PBS twice. The tubes were flash frozen in liquid nitrogen and stored at –80°C until extraction. RNA extractions were performed by using TRIzol reagent according to the kit instructions (Invitrogen Life Technologies). The quality test of extracted RNA from all time points was assessed using a NanoDrop spectrophotometer (Thermo Scientific) and an Agilent RNA 6000 Nano kit on Bioanalyzer 2100 (Agilent Technologies). Only the RNA samples with a concentration of more than 200 ng/μl, 1.8 < *A*_260_/*A*_280_ < 2.0, and the RNA integrity (RIN) value higher than or equal to 8 were further used to construct cDNA libraries. For depletion of rRNA, a Ribo-Zero rRNA removal kit (Illumina, San Diego, CA, USA) was used. Random oligonucleotides and SuperScript III were used to synthesize the first-strand cDNA. Second-strand cDNA synthesis was subsequently performed using DNA polymerase I and RNase H. The remaining overhangs were converted into blunt ends via exonuclease/polymerase activities, and the enzymes were removed. To select cDNA fragments of the preferred 300-bp length, the library fragments were purified using the AMPure XP system (Beckman Coulter, Beverly, CA, USA). DNA fragments with ligated adaptor molecules on both ends were selectively enriched using Illumina PCR Primer Cocktail in a 15-cycle PCR. Products were purified (AMPure XP system) and quantified using the Agilent High Sensitivity DNA assay on a Bioanalyzer 2100 system (Agilent). The sequencing library was then sequenced on a NextSeq 500 platform (Illumina) by Shanghai Personal Biotechnology Co., Ltd. Images from the instrument were processed using the manufacturer’s software to generate FASTQ sequence files.

### Read alignment from RNA-seq.

Raw transcriptome sequencing (RNA-seq) reads were preprocessed using the in-house Perl scripts and sickle software (version 1.200) (62) with arguments “pe –t illumina -l 50 –q 5.” The preprocessed reads for each library were aligned to the combined transcripts of strain TW11 and phage JD032 using Bowtie2 (version 2.1.0) (63). Gene-level read counts were summarized via SAMtools (version 0.1.18) (64). The rRNA genes as well as the genes undetected (without any read) were removed, generating a total of 3,835 genes for further analysis, including 3,781 host genes and 54 phage genes.

### Differential gene expression analysis.

The RUV method in the Bioconductor package RUVSeq (version 1.0.0) (http://www.bioconductor.org/packages/release/bioc/html/RUVSeq.html) was used to normalize the raw counts of the genes and identify differentially expressed genes (DEGs) between phage-infected TW11 strains at any time point and the uninfected control. The RUV method takes negative-control samples for which the covariates of interest are constant. Here, a DEG met the following conditions: average expression levels of the gene in a group (infected or control) with more than five reads per kilobase per million mapped reads (RPKM), false discovery rate (FDR, an adjusted *P* value after multiple testing of Benjamini-Hochberg [BH]) < 0.01 and fold change (FC) (infected strain/control) of ≥2 (upregulated) or ≤−2 (downregulated).

### Hierarchical cluster analysis.

The DEGs between the phage-infected TW11 strain and the control at each time point were clustered using the hclust function in the stats package in R software (https://www.r-project.org/). The heatmap.2 function in the gplots package was used to plot a heatmap for the DEGs at any time point.

### KEGG enrichment analysis.

Functional annotation of TW11 genes was conducted using the KEGG Automatic Annotation Server (https://www.genome.jp/tools/kaas/). The resulting KO (KEGG Orthology) and KEGG pathways were further subjected to enrichment analysis of DEGs. The phyper function in the R stats package was used to enrich the KEGG pathway. Here, enrichment analysis of the upregulated and downregulated genes at any time point was executed. A KEGG pathway with an adjusted *P* value of <0.05 and gene number of ≥3 was defined as significant.

### Quantitative reverse transcription-PCR.

To validate the RNA-seq data, quantitative reverse transcription-PCR (qRT-PCR) was performed on four phage genes and eight host genes. Primers for RT-qPCR are listed in Table S8 in the supplemental material. The genomic DNA (gDNA) was removed by incubating with DNase I Recombinant (Sigma-Aldrich) for 30 min at 25°C. cDNA synthesis was performed on 1 μg of total RNA with a RevertAid First Strand cDNA Synthesis kit (Thermo Scientific) according to the manufacturer’s instructions. qRT-PCR was performed using an ABI 7500 real-time PCR system (Applied Biosystems) with FastStart universal SYBR green master mix (Roche). The following cycling conditions were used: 10 min at 95°C and 40 cycles, with 1 cycle consisting of 15 s at 95°C and 60 s at 60°C. All samples from three biological replicates of each time point were amplified in a 96-well plate. The relative expression levels were normalized to the expression of glutamate dehydrogenase (*gluD*) (65).

10.1128/mSystems.00017-20.10 TABLE S8 Primers used in this study. Download Table S8, PDF file, 0.01 MB. Copyright © 2020 Li et al. 2020 Li et al. This content is distributed under the terms of the Creative Commons Attribution 4.0 International license.

### Data availability.

The whole genome of phage JD032 was deposited at GenBank under accession number MK473382. The whole-genome sequence of TW11 was deposited at NCBI database under accession number PRJNA558841. The raw reads of RNA-seq (FASTQ files) are publicly available at NCBI database under accession number PRJNA559590.
