# Supplementary material for: Isolation and Characterization of the Novel Phage JD032 and Global Transcriptomic Response during JD032 Infection of Clostridioides difficile Ribotype 078
Source: mSystems. 2020 May 5;5(3):e00017-20. doi: 10.1128/mSystems.00017-20 (PMC7205517; doi:10.1128/mSystems.00017-20)
Supplement: TABLE S1 [file mSystems.00017-20-st001.pdf]

Table S1. Gene annotation and expression data of phage JD032

Query_name	Gene	From	To	Product	Log2 RPKM at 30min	Log2 RPKM at 45min	Log2 RPKM at 75min	Log2 RPKM at 135min	Cluster
contig1_1	JD032_orf001	347	54	HNH endonuclease	8.377	12.474	13.511	13.022	2
contig1_2	JD032_orf002	637	347	hypothetical protein	7.312	12.178	13.201	12.608	2
contig1_3	JD032_orf003	1337	834	Probable integrase/recombinase YoeC OS	9.013	13.553	14.653	13.676	2
contig1_4	JD032_orf004	2048	1581	Putative RNA polymerase sigma factor	10.691	11.164	10.090	11.195	1
contig1_5	JD032_orf005	2612	2151	Putative RNA polymerase sigma factor	11.799	12.257	11.052	11.833	1
contig1_6	JD032_orf006	3120	2860	hypothetical protein	12.230	13.056	11.579	11.858	1
contig1_7	JD032_orf007	3407	3123	hypothetical protein	12.421	13.110	11.840	12.283	1
contig1_8	JD032_orf008	3552	3397	hypothetical protein	11.912	12.583	11.434	11.982	1
contig1_9	JD032_orf009	3945	3565	Single-stranded DNA-binding protein	12.502	13.035	12.034	12.617	1
contig1_10	JD032_orf010	4218	4003	hypothetical protein	12.114	12.621	11.621	12.242	1
contig1_11	JD032_orf011	4486	4211	hypothetical protein	12.390	13.073	12.083	12.373	1
contig1_12	JD032_orf012	4830	4498	hypothetical protein	12.721	13.467	12.567	12.782	1
contig1_13	JD032_orf013	4493	4832	hypothetical protein	12.967	13.809	12.903	12.970	1
contig1_14	JD032_orf014	5525	5091	hypothetical protein	14.455	15.543	14.958	13.880	1
contig1_15	JD032_orf015	5838	5590	hypothetical protein	12.788	13.994	13.235	12.326	1
contig1_16	JD032_orf016	5873	6262	XRE family transcriptional regulator	14.086	15.150	14.994	14.375	1
contig1_17	JD032_orf017	6417	6283	protein of unknown function	6.672	8.578	9.373	11.087	2
contig1_18	JD032_orf018	8925	6778	DEAD/DEAH box helicase (Type III restriction enzyme)	8.569	9.936	10.085	10.948	2
contig1_19	JD032_orf019	9369	10091	3'-5' exonuclease(DNA polymerase III PolC-type)	8.307	11.310	11.656	11.544	2
contig1_20	JD032_orf020	10937	11119	hypothetical protein	9.550	10.641	10.897	11.580	2
contig1_21	JD032_orf021	11406	12215	ParA family protein	10.451	11.826	12.312	12.131	2
contig1_22	JD032_orf022	12216	12551	hypothetical protein	10.827	12.180	12.668	12.563	2
contig1_23	JD032_orf023	12696	12995	hypothetical protein	10.858	12.540	13.039	13.068	2
contig1_24	JD032_orf024	13709	13239	hypothetical protein	10.781	11.420	12.402	11.903	2
contig1_25	JD032_orf025	14421	13786	putative regulatory protein	8.504	10.515	11.829	11.795	3
contig1_26	JD032_orf026	14882	14433	hypothetical protein	7.238	9.940	11.308	11.348	3
contig1_27	JD032_orf027	15032	15241	hypothetical protein	8.909	10.290	11.159	11.769	3
contig1_28	JD032_orf028	16107	15268	putative endolysin protein	7.554	10.092	12.067	12.034	3
contig1_29	JD032_orf029	16364	16107	putative holin protein	5.905	10.037	11.927	11.717	3
contig1_30	JD032_orf030	16614	16384	membrane protein	7.515	10.579	12.349	12.519	3
contig1_31	JD032_orf031	16835	16653	hypothetical protein	6.095	10.150	11.932	12.063	3
contig1_32	JD032_orf032	17128	16835	hypothetical protein	5.224	10.559	12.360	11.878	3
contig1_33	JD032_orf033	18839	17142	hypothetical protein	5.962	10.031	11.717	11.661	3
contig1_34	JD032_orf034	19636	18857	Probable tail fiber protein	6.690	10.340	11.661	11.674	3

contig1_35	JD032_orf035	20254	19637	Phage-like element PBSX protein XkdU	5.945	10.378	11.643	11.544	3
contig1_36	JD032_orf036	21306	20254	baseplate protein J	6.370	10.788	11.991	11.783	3
contig1_37	JD032_orf037	21727	21299	Protein of unknown function (DUF2634)	5.953	10.779	12.023	11.858	3
contig1_38	JD032_orf038	22053	21727	conserved hypothetical protein (XkdS-like)	6.136	10.571	11.718	11.791	3
contig1_39	JD032_orf039	23616	22069	putative cell wall hydrolase protein	6.711	10.950	11.963	11.916	3
contig1_40	JD032_orf040	24467	23829	peptidoglycan-binding protein LysM	6.405	11.242	12.306	12.046	3
contig1_41	JD032_orf041	26221	24467	putative tail tape measure protein	7.276	11.597	12.622	12.205	3
contig1_42	JD032_orf042	26393	26214	hypothetical protein	7.036	11.232	12.257	12.262	3
contig1_43	JD032_orf043	26788	16405	phage XkdN-like family protein	6.602	11.319	12.311	12.168	3
contig1_44	JD032_orf044	27284	26835	Phage tail tube protein	7.534	11.549	13.523	13.082	3
contig1_45	JD032_orf045	28365	27298	Tail sheath protein	6.842	11.827	13.405	12.909	3
contig1_46	JD032_orf046	28798	28382	hypothetical protein	6.539	11.248	12.742	12.630	3
contig1_47	JD032_orf047	29223	28810	hypothetical protein	6.397	11.560	13.233	12.948	3
contig1_48	JD032_orf048	29554	29216	head-tail adaptor protein	6.621	11.397	12.944	12.710	3
contig1_49	JD032_orf049	29845	29558	phage gp6-like head-tail connector protein	6.531	11.576	13.092	12.819	3
contig1_50	JD032_orf050	31123	29855	phage major capsid protein	7.245	11.631	13.104	12.852	3
contig1_51	JD032_orf051	31853	31116	ATP-dependent Clp protease proteolytic subunit	6.582	11.424	12.818	12.527	3
contig1_52	JD032_orf052	33013	31859	phage portal protein	7.004	12.231	13.342	12.636	2
contig1_53	JD032_orf053	34678	33029	Putative terminase large subunit	7.288	12.333	13.402	12.872	2
contig1_54	JD032_orf054	34914	34675	hypothetical protein	8.010	12.615	13.651	13.323	2
