# Supplementary material for: Isolation and Characterization of the Novel Phage JD032 and Global Transcriptomic Response during JD032 Infection of Clostridioides difficile Ribotype 078
Source: mSystems. 2020 May 5;5(3):e00017-20. doi: 10.1128/mSystems.00017-20 (PMC7205517; doi:10.1128/mSystems.00017-20)
Supplement: TABLE S2 [file mSystems.00017-20-st002.pdf]

Table S2. General features and accession number of phages used in this study

Phage	Family	Genome size(bp)	Number of CDS[a]	Accession No.
phiCD119	M	53,325	97	AY855346.1
phiC2	M	56,538	97	DQ466086.1
phiCD27	M	50,930	73	NC_011398.1
phiCD6356	S	37,664	61	NC_015262.1
phiCD38-2	S	41,090	52	HM568888.1
phiMMP02	M	48,396	72	JX145341.1
phiMMP04	M	31,674	48	JX145342.1
phiCDHM1	M	54,279	82	NC_024144.1
phiCDHM13	M	33,596	50	HG796225.1
phiCDHM14	M	33,596	48	LK985321.1
phiCDHM19	M	54,295	89	LK985322.1
phiCD211[b]	S	131,326	178	NC_029048.1
phiCDIF1296T[b]	S	131,326	178	CP011970.1
phiCD24-1	S	44,129	60	LN681534.1
phiCD111	S	41,560	54	LN681535.1
phiCD146	S	41,507	51	LN681536.1
phiMMP01	M	44,461	79	LN681541.1
phiMMP03	M	52,261	91	LN681542.1
phiCD481-1	M	32,846	49	LN681538.1
phiCD505	M	49,316	72	LN681539.1
phiCD506	M	33,274	51	LN681540.1
phiCDHM11	M	32,000	50	HG798901.1
phiCDKM9	M	49,822	76	KX228399
phiCDKM15	M	50,605	80	KX228400
phiSemix9P1	N/A[c]	56,606	36	KX905163.1

[a]CDSs were predicted by PHASTER (http://phaster.ca/)

[b]phiCDIF1296T and phiCD211 are the same phage.

[c]N/A represents that the morphology has not been observed under TEM
