# Supplementary material for: Isolation and Characterization of the Novel Phage JD032 and Global Transcriptomic Response during JD032 Infection of Clostridioides difficile Ribotype 078
Source: mSystems. 2020 May 5;5(3):e00017-20. doi: 10.1128/mSystems.00017-20 (PMC7205517; doi:10.1128/mSystems.00017-20)
Supplement: TABLE S3 [file mSystems.00017-20-st003.pdf]

Table S3. Predicted CRISPR-Cas systems of *C. difficile* TW11[a].

Locus	Start in bp	Stop in bp	Number of cas	Number of	Cas (sub)type	Genes
Locus.1	769375	780980	9	31	WYL, cas6, cas8b2, cas7, cas5, cas3, cas4,acs1, cas2	TW11_00692-TW11_00792
Locus.2	1068945	1071781	0	43		TW11_00936
Locus.3	1428489	1434977	4	0	cas6, cas7, cas5, cas3	TW11_01257-TW11_01261
Locus.4	1786185	1797838	7	38	cas6, cas8b1, cas7b, cas5, cas3, cas4, cas1	TW11_01580-TW11_01587
Locus.5	1905827	1906548	0	11		None
Locus.6	2318759	2323229	1	17	c2c9_V_U4	None
Locus.7	2802314	2802912	0	9		None

[a]CRISPR-Cas systems were predicted by CRISPRminer (http://www.microbiome-bigdata.com/CRISPRminer/)
