# Supplementary material for: Isolation and Characterization of the Novel Phage JD032 and Global Transcriptomic Response during JD032 Infection of Clostridioides difficile Ribotype 078
Source: mSystems. 2020 May 5;5(3):e00017-20. doi: 10.1128/mSystems.00017-20 (PMC7205517; doi:10.1128/mSystems.00017-20)
Supplement: TABLE S4 [file mSystems.00017-20-st004.pdf]

Table S4. Predicted restriction modification (RM) system genes and their transcript levels at four time points during phage JD032 infection[a].

Gene	Annotation	Type	30min[b] p.i		45min[b] p.i		75min[b] p.i		135min[b] p.i	
			log_2FC	FDR	log_2FC	FDR	log_2FC	FDR	log_2FC	FDR
TW11_0859	SAM-dependent methyltransferase	2	-0.07	0.915	-0.12	0.542	0.02	0.942	-0.51	0.035
TW11_1134	ribulose-phosphate 3-epimerase	-1	0.44	0.228	0.05	0.802	-0.23	0.227	-0.45	0.079
TW11_1493	transcriptional regulator	2	0.08	0.915	0.47	0.015	0.68	0.002	0.62	0.114
TW11_1588	transcriptional regulator	2	-0.11	0.870	-0.23	0.380	-0.82	0.001	0.21	0.560
TW11_1826	adenine methyltransferase	2	0.37	0.722	0.89	0.093	0.19	0.718	-0.67	0.132
TW11_2404	DNA translocase FtsK	2	-0.28	0.510	-0.07	0.756	-0.16	0.512	0.49	0.065
TW11_2449	cysteine desulfurase NifS	2	0.16	0.742	-0.25	0.140	-0.57	0.000	-0.38	0.129
TW11_2454	type I DNA topoisomerase	2	-0.58	0.080	-0.81	0.000	**-1.03**	**0.000**	-0.41	0.150
TW11_3417	chaperonin GroEL	2	-0.05	0.953	0.02	0.963	-0.02	0.941	0.18	0.628

[a]RM systems were predicted by REBASE (http://rebase.neb.com/rebase/rebase.html)

[b]Positive and negative values indicate an increase and decrease in transcript levels during infection with significant differences shown in red and blue, respect
