# Supplementary material for: Isolation and Characterization of the Novel Phage JD032 and Global Transcriptomic Response during JD032 Infection of Clostridioides difficile Ribotype 078
Source: mSystems. 2020 May 5;5(3):e00017-20. doi: 10.1128/mSystems.00017-20 (PMC7205517; doi:10.1128/mSystems.00017-20)
Supplement: TABLE S5 [file mSystems.00017-20-st005.pdf]

Table S5. Predicted toxin-antitoxin (TA) system genes and their transcript levels at four time points during phage JD032 infection[a].

T/A system	Gene	Family	Domain	Annotation	30min[b] p.i		45min[b] p.i		75min[b] p.i		135min[b] p.i	
					log_2FC	FDR	log_2FC	FDR	log_2FC	FDR	log_2FC	FDR
AT	TW11_0231	RHHlike_domain	NULL	antitoxin	0.25	0.528	0.18	0.320	0.05	0.825	-0.30	0.252
T	TW11_0232	MazFlike_domain	NULL	type II toxin-antitoxin system PemK/MazF family toxin	0.21	0.605	0.16	0.375	0.06	0.788	-0.41	0.094
AT	TW11_1205	NULL	pfam00440	TetR/AcrR family transcriptional regulator	0.15	0.777	0.25	0.285	0.91	0.000	**2.00**	**0.000**
T	TW11_1204	NULL	pfam12568	MFS transporter	0.18	0.743	0.57	0.011	**1.11**	**0.000**	**1.87**	**0.000**
AT	TW11_2369	Xrelike_domain	NULL	XRE family transcriptional regulator	0.99	0.000	**1.12**	**0.000**	**1.46**	**0.000**	**1.53**	**0.000**
T	TW11_2370	COG2856like_domain	NULL	ImmA/IrrE family metallo-endopeptidase	0.66	0.036	0.72	0.000	**1.06**	**0.000**	0.44	0.110
AT	TW11_2450	NULL	cd00090	Rrf2 family transcriptional regulator	-0.05	0.940	-0.58	0.001	**-1.28**	**0.000**	-0.91	0.000
T	TW11_2451	NULL	pfam12746	N-acetyltransferase	-0.20	0.708	-0.16	0.490	-0.46	0.029	0.36	0.252
AT	TW11_2518	NULL	pfam01047	N-acetyltransferase	0.29	0.557	0.51	0.007	0.44	0.062	0.37	0.317
T	TW11_2519	NULL	COG1246	N-acetyltransferase	0.18	0.705	0.50	0.002	0.49	0.006	0.34	0.235
AT	TW11_2553	NULL	pfam08220	transcription factor FapR	-0.27	0.512	-0.32	0.067	-0.30	0.121	-0.16	0.599
T	TW11_2552	NULL	COG1569	phosphate acyltransferase	-0.19	0.720	-0.34	0.044	-0.37	0.077	-0.08	0.814
AT	TW11_2815	NULL	pfam01022	Iron dependent repressor DNA-binding domain-containing protein	0.16	0.767	0.01	0.973	0.17	0.454	**1.13**	**0.000**
T	TW11_2814	NULL	PRK07757	N-acetyltransferase	0.31	0.427	0.21	0.289	0.51	0.006	0.87	0.001

[a]TA systems were predicted by TA finder (http://202.120.12.133/TAfinder/index.php)

[b]Positive and negative values indicate an increase and decrease in transcript levels during infection with significant differences shown in red and blue, respectively.
