# Supplementary material for: Isolation and Characterization of the Novel Phage JD032 and Global Transcriptomic Response during JD032 Infection of Clostridioides difficile Ribotype 078
Source: mSystems. 2020 May 5;5(3):e00017-20. doi: 10.1128/mSystems.00017-20 (PMC7205517; doi:10.1128/mSystems.00017-20)
Supplement: TABLE S6 [file mSystems.00017-20-st006.pdf]

Table S6. DEGs in *C. difficile* strain TW11 upon phage JD032 infection.

Gene	Annotation	30min p. i		45min p. i		75min p. i		135min p. i	
		log_2FC	FDR	log_2FC	FDR	log_2FC	FDR	log_2FC	FDR
TW11_0015	sulfurtransferase-like selenium metabolism	-0.32	0.4406	-0.85	0.0000	**-1.01**	**0.0000**	-0.84	0.0003
TW11_0016	hypothetical protein	-0.37	0.3655	-0.88	0.0000	**-1.15**	**0.0000**	**-1.06**	**0.0000**
TW11_0017	hypothetical protein	-0.41	0.3113	-0.99	0.0000	**-1.13**	**0.0000**	-0.84	0.0006
TW11_0027	hypothetical protein	-0.87	0.0323	-0.95	0.0000	**-1.05**	**0.0000**	-0.64	0.0322
TW11_0043	hypothetical protein	0.42	0.4151	0.39	0.1787	0.18	0.5882	**-1.06**	**0.0002**
TW11_0044	amidohydrolase/deacetylase family	0.42	0.3846	0.49	0.0717	0.43	0.1155	**-1.09**	**0.0001**
TW11_0045	SelA-like pyridoxal phosphate-dependent enzyme	0.05	0.9502	0.24	0.4230	-0.05	0.8766	**-1.23**	**0.0000**
TW11_0048	LysR family transcriptional regulator	0.91	0.0022	**1.39**	**0.0000**	**1.15**	**0.0000**	0.67	0.0111
TW11_0050	flavodoxin family protein	-0.24	0.6334	-0.16	0.5538	-0.17	0.4875	**-1.06**	**0.0002**
TW11_0052	hypothetical protein	-0.14	0.8120	-0.27	0.2381	-0.18	0.4577	**-1.10**	**0.0001**
TW11_0071	VOC family protein	0.71	0.0198	**1.02**	**0.0000**	0.65	0.0002	0.42	0.0941
TW11_0073	potassium/proton antiporter	-0.85	0.0033	-0.36	0.0583	-0.13	0.5904	**1.01**	**0.0001**
TW11_0074	MerR family transcriptional regulator	-0.56	0.0798	-0.22	0.2719	-0.06	0.8195	**1.27**	**0.0000**
TW11_0076	staygreen family protein	-0.72	0.0117	-0.71	0.0001	-0.86	0.0000	**-1.14**	**0.0000**
TW11_0077	MATE family efflux transporter	-0.85	0.0025	-0.99	0.0000	-0.85	0.0000	**-1.11**	**0.0000**
TW11_0080	ABC transporter ATP-binding protein	-0.13	0.7806	-0.63	0.0000	-0.95	0.0000	**-1.04**	**0.0000**
TW11_0097	orotidine-5'-phosphate decarboxylase	0.18	0.7344	0.15	0.5305	0.96	0.0000	**1.50**	**0.0000**
TW11_0098	carbamoyl-phosphate synthase small subunit	-0.10	0.8812	0.16	0.4381	0.76	0.0001	**1.34**	**0.0000**
TW11_0126	peptidoglycan-binding protein	**-1.35**	**0.0000**	-0.60	0.0007	-0.53	0.0041	0.07	0.8475
TW11_0146	hypothetical protein	**-2.34**	**0.0000**	**-1.73**	**0.0000**	**-1.89**	**0.0000**	**-2.18**	**0.0000**
TW11_0147	arginine decarboxylase	-0.63	0.0534	-0.90	0.0000	-0.97	0.0000	**-1.05**	**0.0000**
TW11_0153	HD domain-containing protein	-0.11	0.8745	-0.27	0.1162	-0.54	0.0125	**-1.01**	**0.0001**
TW11_0156	spore maturation protein A	0.10	0.9164	0.04	0.9214	0.19	0.5845	**-1.72**	**0.0000**
TW11_0158	methionine--tRNA ligase	-0.07	0.9318	-0.95	0.0000	**-1.29**	**0.0000**	-0.98	0.0006
TW11_0160	phosphonate C-P lyase system protein PhnG	-1.43	0.2150	0.88	0.1254	**1.82**	**0.0001**	**3.17**	**0.0000**
TW11_0169	M42 glutamyl aminopeptidase	**1.20**	**0.0000**	**1.15**	**0.0000**	0.92	0.0000	0.72	0.0029
TW11_0203	UTP--glucose-1-phosphate uridylyltransferase	**1.16**	**0.0000**	**1.28**	**0.0000**	0.91	0.0000	0.76	0.0035
TW11_0204	hypothetical protein	0.66	0.0905	**1.28**	**0.0000**	0.72	0.0010	0.00	0.9967
TW11_0216	dCMP deaminase	-0.48	0.2611	-0.69	0.0000	-0.80	0.0000	**-1.00**	**0.0000**
TW11_0219	ATP synthase F0 subunit A	-0.73	0.0092	**-1.04**	**0.0000**	**-1.19**	**0.0000**	-0.79	0.0010
TW11_0221	ATP synthase subunit B	-0.89	0.0110	**-1.32**	**0.0000**	**-1.39**	**0.0000**	-0.62	0.0416
TW11_0222	ATP synthase subunit delta	-0.82	0.0437	**-1.27**	**0.0000**	**-1.42**	**0.0000**	-0.70	0.0285
TW11_0223	ATP synthase subunit alpha	-0.70	0.1122	**-1.11**	**0.0000**	**-1.34**	**0.0000**	-0.66	0.0342
TW11_0224	ATP synthase F1 subunit gamma	-0.66	0.1986	**-1.18**	**0.0000**	**-1.43**	**0.0000**	-0.69	0.0344
TW11_0225	F0F1 ATP synthase subunit beta	-0.39	0.4874	-0.71	0.0005	**-1.09**	**0.0000**	-0.64	0.0422

TW11_0243	SIS domain-containing protein	-0.18	0.7591	-0.37	0.1512	-0.72	0.0089	**-1.39**	**0.0012**
TW11_0244	ketose-bisphosphate aldolase	-0.44	0.3357	-0.14	0.6116	-0.54	0.0287	**-1.10**	**0.0002**
TW11_0246	peptidase M42	-0.02	0.9840	-0.04	0.9198	-0.06	0.8616	**-1.31**	**0.0000**
TW11_0271	precorrin-6A reductase	-0.55	0.2560	**-1.03**	**0.0000**	**-1.03**	**0.0000**	-0.78	0.0115
TW11_0273	hydroxymethylbilane synthase	-0.62	0.1933	**-1.06**	**0.0000**	**-1.02**	**0.0000**	**-1.01**	**0.0003**
TW11_0274	uroporphyrinogen-III C-methyltransferase	-0.64	0.1058	-0.99	0.0000	**-1.01**	**0.0000**	**-1.09**	**0.0000**
TW11_0280	ABC transporter ATP-binding protein	**-1.21**	**0.0002**	**-1.06**	**0.0000**	-0.92	0.0002	-0.81	0.0153
TW11_0281	sugar ABC transporter permease	**-1.52**	**0.0000**	**-1.28**	**0.0000**	**-1.30**	**0.0000**	**-1.07**	**0.0001**
TW11_0282	carbohydrate ABC transporter permease	**-1.31**	**0.0002**	**-1.68**	**0.0000**	**-1.53**	**0.0000**	**-1.18**	**0.0003**
TW11_0283	ABC transporter substrate-binding protein	**-1.13**	**0.0002**	**-1.12**	**0.0000**	**-1.20**	**0.0000**	**-1.23**	**0.0000**
TW11_0302	ATP-dependent 6-phosphofructokinase	-0.87	0.0372	**-1.33**	**0.0000**	**-1.54**	**0.0000**	**-1.16**	**0.0005**
TW11_0303	pyruvate kinase	-0.80	0.0347	**-1.02**	**0.0000**	**-1.30**	**0.0000**	**-1.40**	**0.0000**
TW11_0305	hypothetical protein	0.16	0.8018	0.85	0.0000	**1.05**	**0.0000**	-0.22	0.5378
TW11_0319	transcriptional regulator	0.03	0.9673	0.31	0.1934	0.54	0.0390	**1.22**	**0.0002**
TW11_0325	glycosyl transferase	-0.54	0.2419	-0.68	0.0105	-0.82	0.0011	**-1.06**	**0.0034**
TW11_0330	hypothetical protein	-0.74	0.0125	**-1.45**	**0.0000**	**-2.06**	**0.0000**	**-2.17**	**0.0000**
TW11_0342	MFS transporter	0.21	0.6334	0.66	0.0001	**1.58**	**0.0000**	**1.13**	**0.0000**
TW11_0344	helix-turn-helix domain-containing protein	0.18	0.8030	0.80	0.0012	**2.62**	**0.0000**	**1.28**	**0.0001**
TW11_0345	replication initiation factor domain-containing	0.00	0.9987	0.86	0.0000	**2.64**	**0.0000**	**1.86**	**0.0000**
TW11_0346	hypothetical protein	0.32	0.4623	0.86	0.0000	**2.68**	**0.0000**	**2.45**	**0.0000**
TW11_0347	transcriptional regulator	0.23	0.6164	0.83	0.0000	**1.64**	**0.0000**	0.98	0.0001
TW11_0370	type II secretion system protein	-0.95	0.0110	**-1.06**	**0.0000**	-0.76	0.0042	-0.89	0.0051
TW11_0377	oxidoreductase	-0.67	0.0781	-0.91	0.0000	**-1.03**	**0.0000**	-0.83	0.0005
TW11_0378	Na/Pi cotransporter family protein	**-1.24**	**0.0000**	-0.92	0.0000	-0.97	0.0000	**-1.28**	**0.0000**
TW11_0380	protease	0.20	0.6853	0.27	0.1063	0.42	0.0156	**1.19**	**0.0000**
TW11_0381	glycyl-radical enzyme activating protein	-0.28	0.5640	**-1.51**	**0.0000**	**-2.18**	**0.0000**	**-2.17**	**0.0000**
TW11_0382	glycyl-radical enzyme activating protein	-0.12	0.8235	-0.94	0.0000	**-1.63**	**0.0000**	**-1.94**	**0.0000**
TW11_0383	pyrroline-5-carboxylate reductase	0.28	0.4899	-0.27	0.1540	-0.87	0.0000	**-1.54**	**0.0000**
TW11_0385	PTS mannose/fructose/sorbose transporter subunit	0.19	0.8194	0.56	0.1066	0.26	0.5119	**-1.10**	**0.0009**
TW11_0386	PTS mannose transporter subunit IIA	-0.51	0.4976	-0.20	0.6919	-0.17	0.6955	**-1.12**	**0.0019**
TW11_0387	PTS sugar transporter subunit IIC	-0.47	0.3510	-0.31	0.3358	-0.45	0.1205	**-1.01**	**0.0015**
TW11_0397	DNA-binding response regulator	**-1.13**	**0.0016**	**-1.14**	**0.0000**	-0.83	0.0012	**-1.87**	**0.0000**
TW11_0398	histidine kinase	**-1.31**	**0.0000**	**-1.16**	**0.0000**	-0.94	0.0001	**-1.93**	**0.0000**
TW11_0399	DNA-binding response regulator	**-1.07**	**0.0002**	-0.94	0.0000	-0.74	0.0003	**-1.93**	**0.0000**
TW11_0401	phosphate ABC transporter permease subunit PstC	-0.34	0.5286	0.34	0.1617	**2.07**	**0.0000**	**3.86**	**0.0000**
TW11_0402	phosphate ABC transporter, permease protein	**-1.08**	**0.0027**	-0.60	0.0166	0.38	0.1386	**2.67**	**0.0000**
TW11_0403	phosphate ABC transporter ATP-binding protein	**-1.18**	**0.0008**	-0.86	0.0002	-0.57	0.0170	**1.57**	**0.0000**
TW11_0404	phosphate transport system regulatory protein	-0.96	0.0068	-0.78	0.0018	-0.66	0.0150	**1.05**	**0.0014**

TW11_0406	iron hydrogenase	-0.32	0.5663	0.08	0.8012	-0.08	0.7874	**-1.32**	**0.0000**
TW11_0408	valine--tRNA ligase	-0.13	0.8598	-0.93	0.0000	**-1.15**	**0.0000**	-0.64	0.0336
TW11_0411	bifunctional folylpolyglutamate	0.11	0.8275	0.96	0.0000	**1.26**	**0.0000**	0.14	0.6595
TW11_0417	proline reductase-associated electron transfer	-0.06	0.9241	-0.40	0.0422	-0.41	0.0743	**-1.30**	**0.0000**
TW11_0418	cell surface protein	-0.19	0.6909	**-1.22**	**0.0000**	**-1.42**	**0.0000**	**-1.03**	**0.0000**
TW11_0420	D-proline reductase (dithiol) proprotein PrdA	-0.59	0.2856	**-1.40**	**0.0000**	**-1.53**	**0.0000**	**-2.12**	**0.0000**
TW11_0421	hypothetical protein	-0.66	0.2856	**-1.48**	**0.0000**	**-1.47**	**0.0000**	**-2.03**	**0.0000**
TW11_0422	proline reductase	-0.67	0.2143	**-1.26**	**0.0000**	**-1.22**	**0.0000**	**-2.07**	**0.0000**
TW11_0423	proline reductase	-0.58	0.2732	**-1.01**	**0.0000**	-0.99	0.0000	**-1.87**	**0.0000**
TW11_0424	proline reductase cluster protein PrdD	-0.62	0.2585	**-1.24**	**0.0000**	-0.93	0.0004	**-2.27**	**0.0000**
TW11_0425	D-proline reductase	-0.68	0.2226	**-1.31**	**0.0000**	**-1.01**	**0.0001**	**-2.22**	**0.0000**
TW11_0426	D-proline reductase (dithiol)	-0.40	0.4380	-0.93	0.0000	-0.85	0.0018	**-1.90**	**0.0000**
TW11_0427	proline racemase	0.01	0.9968	-0.51	0.0215	-0.58	0.0148	**-1.54**	**0.0000**
TW11_0430	methyltransferase domain-containing protein	0.85	0.0070	**1.59**	**0.0000**	**1.18**	**0.0000**	**1.10**	**0.0000**
TW11_0434	hypoxanthine phosphoribosyltransferase	-0.38	0.4214	-0.81	0.0000	-0.95	0.0000	**-1.29**	**0.0000**
TW11_0441	aspartate-semialdehyde dehydrogenase	-0.18	0.7883	-0.73	0.0002	**-1.05**	**0.0000**	-0.64	0.0380
TW11_0445	small acid-soluble spore protein	**-1.44**	**0.0000**	**-1.51**	**0.0000**	**-1.29**	**0.0000**	-0.82	0.0223
TW11_0446	class I SAM-dependent methyltransferase	**-1.09**	**0.0001**	-0.76	0.0001	-0.91	0.0000	-0.49	0.1070
TW11_0456	MarR family transcriptional regulator	-0.34	0.4858	-0.10	0.7122	0.44	0.0465	**1.78**	**0.0000**
TW11_0457	multidrug transporter MatE	-0.53	0.2035	-0.09	0.7251	0.04	0.8724	**1.14**	**0.0000**
TW11_0460	hypothetical protein	0.90	0.0136	**1.40**	**0.0000**	**1.20**	**0.0000**	**1.46**	**0.0000**
TW11_0461	protein translocase component YidC	**1.05**	**0.0008**	**1.54**	**0.0000**	**1.21**	**0.0000**	**1.05**	**0.0000**
TW11_0466	ABC transporter permease	-0.62	0.1696	-0.50	0.0540	-0.24	0.3667	**-1.58**	**0.0000**
TW11_0489	chlorohydrolase	-0.65	0.0850	-0.99	0.0000	**-1.23**	**0.0000**	**-1.03**	**0.0001**
TW11_0490	purine permease	-0.75	0.0098	-0.91	0.0000	**-1.02**	**0.0000**	**-1.10**	**0.0000**
TW11_0491	diguanylate cyclase	-0.68	0.0247	-0.86	0.0000	-0.77	0.0001	**-1.30**	**0.0000**
TW11_0495	transcriptional regulator	**-1.63**	**0.0000**	**-2.21**	**0.0000**	**-2.50**	**0.0000**	**-2.73**	**0.0000**
TW11_0496	type I glyceraldehyde-3-phosphate dehydrogenase	**-1.40**	**0.0000**	**-2.06**	**0.0000**	**-2.52**	**0.0000**	**-2.89**	**0.0000**
TW11_0497	phosphoglycerate kinase	**-1.66**	**0.0000**	**-2.30**	**0.0000**	**-2.52**	**0.0000**	**-2.25**	**0.0000**
TW11_0498	triose-phosphate isomerase	**-1.05**	**0.0033**	**-1.67**	**0.0000**	**-1.79**	**0.0000**	**-1.53**	**0.0000**
TW11_0499	2,3-bisphosphoglycerate-independent	-1.03	0.0108	**-1.71**	**0.0000**	**-1.92**	**0.0000**	**-1.63**	**0.0000**
TW11_0500	phosphopyruvate hydratase	-0.65	0.0227	**-1.03**	**0.0000**	**-1.24**	**0.0000**	**-1.51**	**0.0000**
TW11_0501	MarR family transcriptional regulator	-0.99	0.0001	**-1.07**	**0.0000**	-0.93	0.0000	**-1.01**	**0.0000**
TW11_0515	ATP-binding protein	0.50	0.1033	**1.17**	**0.0000**	**1.25**	**0.0000**	**1.09**	**0.0000**
TW11_0516	phosphoenolpyruvate carboxylase	0.23	0.6669	**1.29**	**0.0000**	**1.41**	**0.0000**	0.62	0.0412
TW11_0517	hypothetical protein	0.28	0.6611	0.97	0.0002	**1.10**	**0.0000**	0.53	0.1979
TW11_0518	hypothetical protein	0.48	0.2329	**1.51**	**0.0000**	**1.50**	**0.0000**	**1.16**	**0.0001**
TW11_0525	membrane protein	0.27	0.5542	**1.71**	**0.0000**	**2.48**	**0.0000**	0.94	0.0018

TW11_0526	XRE family transcriptional regulator	0.23	0.6123	**1.75**	**0.0000**	**2.55**	**0.0000**	0.91	0.0003
TW11_0546	MFS transporter	-0.40	0.4608	-0.41	0.1625	-0.50	0.0743	**-1.04**	**0.0003**
TW11_0583	YfcC family protein	**-1.51**	**0.0000**	-0.72	0.0008	-0.76	0.0004	**-1.09**	**0.0021**
TW11_0590	gamma-glutamyl-gamma-aminobutyrate hydrolase	0.92	0.0004	0.88	0.0000	**1.05**	**0.0000**	-0.08	0.7895
TW11_0591	amino acid permease	0.82	0.0021	0.97	0.0000	**1.32**	**0.0000**	0.19	0.4767
TW11_0592	alpha,alpha-phosphotrehalase	0.75	0.0139	**1.02**	**0.0000**	**1.32**	**0.0000**	0.92	0.0004
TW11_0595	putative cellobiose-phosphate degrading protein	**1.10**	**0.0008**	**1.15**	**0.0000**	**1.32**	**0.0000**	0.85	0.0125
TW11_0597	MurR/RpiR family transcriptional regulator	0.26	0.6123	0.83	0.0000	**1.03**	**0.0000**	0.56	0.0427
TW11_0598	1-phosphofructokinase	0.68	0.0454	0.02	0.9516	0.01	0.9590	**-1.30**	**0.0000**
TW11_0637	peptidase C1	**-1.41**	**0.0000**	-0.78	0.0004	-0.28	0.2349	0.78	0.0077
TW11_0641	CarD family transcriptional regulator	0.71	0.0115	**1.22**	**0.0000**	0.89	0.0000	0.43	0.1279
TW11_0642	MFS transporter	0.03	0.9742	-0.29	0.2073	-0.26	0.2525	**-1.35**	**0.0000**
TW11_0644	TrmB family transcriptional regulator	-0.51	0.2257	**-1.00**	**0.0000**	**-1.14**	**0.0000**	-0.10	0.7684
TW11_0645	thioredoxin	**1.25**	**0.0000**	0.76	0.0005	-0.01	0.9787	-0.44	0.1278
TW11_0646	NAD(P)/FAD-dependent oxidoreductase	**1.70**	**0.0000**	**1.58**	**0.0000**	0.76	0.0000	0.34	0.3135
TW11_0651	hypothetical protein	-0.14	0.7845	-0.38	0.0482	0.63	0.0013	**2.52**	**0.0000**
TW11_0652	4Fe-4S dicluster domain protein	-0.16	0.7632	-0.46	0.0107	0.21	0.3476	**1.89**	**0.0000**
TW11_0657	glucose uptake protein	0.66	0.0529	0.56	0.0053	**1.10**	**0.0000**	**1.11**	**0.0000**
TW11_0658	PRD domain-containing protein	**1.51**	**0.0000**	0.87	0.0000	**1.08**	**0.0000**	0.95	0.0017
TW11_0659	PTS mannose transporter subunit IIAB	**1.85**	**0.0000**	**1.11**	**0.0000**	**1.35**	**0.0000**	**1.63**	**0.0000**
TW11_0660	PTS fructose transporter subunit IIB	**1.83**	**0.0000**	0.99	0.0000	**1.32**	**0.0000**	**1.74**	**0.0000**
TW11_0661	PTS mannose transporter subunit IIAB	**1.60**	**0.0000**	0.89	0.0000	**1.34**	**0.0000**	**1.57**	**0.0000**
TW11_0662	glycosyl hydrolase	**1.53**	**0.0000**	0.83	0.0000	**1.34**	**0.0000**	**1.41**	**0.0000**
TW11_0670	2-keto-3-deoxygluconate permease	-0.33	0.5025	-0.54	0.0272	-0.64	0.0103	**-1.04**	**0.0014**
TW11_0692	WYL domain-containing protein	-0.78	0.0315	**-1.05**	**0.0000**	-0.95	0.0009	-1.19	0.0107
TW11_0693	CRISPR-associated endoribonuclease Cas6	**-1.09**	**0.0055**	-0.96	0.0000	-0.88	0.0003	-0.97	0.0270
TW11_0694	type I-B CRISPR-associated protein Cas8b1/Cst1	**-1.66**	**0.0000**	**-2.10**	**0.0000**	**-1.95**	**0.0000**	**-1.93**	**0.0000**
TW11_0695	type I-B CRISPR-associated protein	**-1.02**	**0.0021**	**-1.34**	**0.0000**	**-1.29**	**0.0000**	**-1.23**	**0.0026**
TW11_0696	type I-B CRISPR-associated protein Cas5	-0.65	0.0781	**-1.10**	**0.0000**	**-1.26**	**0.0000**	-0.94	0.0026
TW11_0697	CRISPR-associated helicase/endonuclease Cas3	-0.82	0.0752	**-1.39**	**0.0000**	**-1.38**	**0.0000**	**-1.06**	**0.0008**
TW11_0698	CRISPR-associated protein Cas4	-0.79	0.0745	**-1.31**	**0.0000**	**-1.48**	**0.0000**	**-1.15**	**0.0002**
TW11_0699	type I-B CRISPR-associated endonuclease Cas1	-0.68	0.0565	**-1.21**	**0.0000**	**-1.07**	**0.0000**	**-1.01**	**0.0003**
TW11_0700	CRISPR-associated endonuclease Cas2	-0.67	0.0338	-0.99	0.0000	**-1.18**	**0.0000**	**-1.16**	**0.0000**
TW11_0702	signal peptidase	0.08	0.9157	0.57	0.0035	**1.01**	**0.0000**	0.60	0.0367
TW11_0704	hypothetical protein	0.67	0.0562	**1.10**	**0.0000**	**1.07**	**0.0000**	**1.13**	**0.0000**
TW11_0711	bifunctional acetaldehyde-CoA/alcohol	**-1.30**	**0.0002**	-1.92	0.0000	-2.38	0.0000	-1.86	0.0006
TW11_0713	tRNA-binding protein	**1.14**	**0.0000**	0.75	0.0000	0.38	0.0586	0.57	0.0322
TW11_0727	putative 2-aminoethylphosphonate ABC transporter	**0.35**	**0.3679**	0.54	0.0015	1.00	0.0000	0.15	0.6115

TW11_0729	trehalase	**1.63**	**0.0001**	**1.60**	**0.0000**	**1.81**	**0.0000**	0.86	0.0139
TW11_0730	PTS trehalose transporter subunit IIBC	**2.13**	**0.0000**	**2.13**	**0.0000**	**2.30**	**0.0000**	**2.03**	**0.0000**
TW11_0731	alpha,alpha-phosphotrehalase	**1.89**	**0.0000**	**1.78**	**0.0000**	**1.97**	**0.0000**	**1.54**	**0.0004**
TW11_0739	ferrichrome ABC transporter substrate-binding	-0.35	0.6279	-0.32	0.3894	-0.07	0.8455	**2.07**	**0.0001**
TW11_0770	polymer-forming cytoskeletal family protein	0.45	0.2807	**1.16**	**0.0000**	**1.20**	**0.0000**	**1.39**	**0.0000**
TW11_0771	polymer-forming cytoskeletal protein	0.32	0.4509	0.95	0.0000	**1.24**	**0.0000**	0.90	0.0003
TW11_0772	hypothetical protein	0.60	0.0553	**1.47**	**0.0000**	**1.69**	**0.0000**	**1.16**	**0.0000**
TW11_0782	amidohydrolase	-0.14	0.8535	0.04	0.9074	-0.14	0.6796	**-1.10**	**0.0002**
TW11_0783	aminobenzoyl-glutamate transporter	-0.56	0.1868	-0.48	0.0472	-0.21	0.4360	**-1.12**	**0.0001**
TW11_0787	SrtB-anchored collagen-binding adhesin	-0.45	0.2075	**-1.09**	**0.0000**	**-1.02**	**0.0000**	-0.73	0.0046
TW11_0791	MarR family transcriptional regulator	0.40	0.2974	0.42	0.0351	0.65	0.0019	**1.39**	**0.0000**
TW11_0803	2-hydroxy-3-oxopropionate reductase	-0.01	0.9968	0.26	0.4789	0.14	0.7107	**-1.23**	**0.0002**
TW11_0821	cell wall-binding protein Cwp9	0.00	0.9968	0.04	0.8901	0.43	0.0186	**1.58**	**0.0000**
TW11_0825	preprotein translocase subunit SecA	-0.38	0.4610	-0.89	0.0000	**-1.09**	**0.0000**	-0.87	0.0012
TW11_0826	cell wall-binding protein Cwp2	-0.14	0.7738	-0.91	0.0000	**-1.20**	**0.0000**	-0.85	0.0003
TW11_0827	PIG-L family deacetylase	-0.34	0.5581	**-1.17**	**0.0000**	**-1.53**	**0.0000**	-0.91	0.0038
TW11_0828	cell wall-binding repeat-containing protein	-0.16	0.8012	-0.68	0.0002	**-1.13**	**0.0000**	-0.50	0.0740
TW11_0836	murein biosynthesis integral membrane protein	-0.99	0.0003	-0.99	0.0000	**-1.28**	**0.0000**	**-1.21**	**0.0000**
TW11_0837	phospho-sugar mutase	-0.95	0.0006	**-1.01**	**0.0000**	**-1.19**	**0.0000**	-0.82	0.0006
TW11_0838	mannose-1-phosphate guanylyltransferase	-0.81	0.0156	-0.95	0.0000	**-1.09**	**0.0000**	-0.68	0.0093
TW11_0845	glycosyltransferase family 2 protein	-0.70	0.0928	**-1.04**	**0.0000**	**-1.05**	**0.0000**	-0.60	0.0470
TW11_0849	NlpC/P60 family protein	0.07	0.9328	0.13	0.5094	0.84	0.0000	**1.50**	**0.0000**
TW11_0860	phospholipase	-0.33	0.3535	-0.37	0.0240	-0.41	0.0203	**-1.34**	**0.0000**
TW11_0861	HPr family phosphocarrier protein	-0.24	0.5854	-0.56	0.0004	-0.74	0.0000	**-1.32**	**0.0000**
TW11_0862	phosphoenolpyruvate--protein phosphotransferase	0.01	0.9827	-0.31	0.0560	-0.40	0.0204	**-1.22**	**0.0000**
TW11_0863	hypothetical protein	-0.54	0.1257	-0.40	0.0499	0.11	0.7428	**1.26**	**0.0009**
TW11_0865	accessory regulator	-0.08	0.9155	0.45	0.0409	0.30	0.2139	**-1.19**	**0.0000**
TW11_0866	accessory regulator	-0.08	0.9116	0.29	0.1436	0.24	0.3203	**-1.34**	**0.0000**
TW11_0867	cyclic lactone autoinducer peptide	0.02	0.9742	0.36	0.0893	0.47	0.0446	**-1.22**	**0.0000**
TW11_0883	membrane protein	-0.02	0.9762	-0.52	0.0413	**-1.43**	**0.0000**	-0.49	0.1141
TW11_0894	serine hydroxymethyltransferase	**1.29**	**0.0000**	**1.35**	**0.0000**	**1.20**	**0.0000**	**1.32**	**0.0000**
TW11_0919	amidohydrolase	**-1.06**	**0.0003**	-0.55	0.0167	-0.23	0.3542	0.01	0.9737
TW11_0926	membrane protein	-0.79	0.0401	-0.24	0.4033	-0.35	0.2205	**-1.11**	**0.0029**
TW11_0933	site-specific integrase	0.16	0.7707	0.98	0.0000	**1.34**	**0.0000**	0.65	0.0229
TW11_0934	integrase	0.25	0.6408	0.95	0.0000	**1.24**	**0.0000**	0.54	0.1335
TW11_0935	hypothetical protein	0.44	0.2501	**1.26**	**0.0000**	**1.43**	**0.0000**	0.30	0.2823
TW11_0937	XRE family transcriptional regulator	-0.14	0.9090	0.43	0.3370	**1.33**	**0.0002**	0.85	0.1116
TW11_0954	membrane protein	**-1.33**	**0.0005**	**-1.31**	**0.0002**	**-1.33**	**0.0000**	**-2.39**	**0.0001**

TW11_0956	cell wall-binding protein Cwp28	-0.24	0.7554	-0.56	0.0316	-0.84	0.0056	**-1.08**	**0.0058**
TW11_0993	F420-0--gamma-glutamyl ligase	-0.27	0.5770	-0.70	0.0000	-0.73	0.0001	**-1.58**	**0.0000**
TW11_0995	hypothetical protein	0.24	0.6517	0.12	0.6758	0.56	0.0246	**1.06**	**0.0005**
TW11_0996	transposase	0.36	0.4090	0.37	0.1228	0.73	0.0012	**1.32**	**0.0000**
TW11_0997	ATP-binding protein	0.05	0.9466	0.19	0.3821	0.74	0.0002	**1.43**	**0.0000**
TW11_0998	XRE family transcriptional regulator	0.34	0.3983	0.47	0.0334	**1.02**	**0.0000**	**1.67**	**0.0000**
TW11_0999	conjugal transfer protein	0.31	0.5709	0.09	0.8108	0.87	0.0006	**1.03**	**0.0027**
TW11_1000	antirestriction protein ArdA	0.41	0.3312	0.52	0.0441	**1.01**	**0.0000**	**1.45**	**0.0000**
TW11_1001	ATP/GTP-binding protein	0.09	0.8985	0.19	0.4904	0.86	0.0000	**1.43**	**0.0000**
TW11_1002	ATP/GTP-binding protein	0.21	0.6489	0.43	0.0362	0.94	0.0000	**1.40**	**0.0000**
TW11_1003	membrane protein	0.26	0.5575	0.47	0.0355	**1.16**	**0.0000**	**1.92**	**0.0000**
TW11_1004	peptidase P60	0.02	0.9775	0.41	0.0821	**1.18**	**0.0000**	**1.69**	**0.0000**
TW11_1005	conjugal transfer protein	0.44	0.2750	0.40	0.1052	**1.15**	**0.0000**	**1.82**	**0.0000**
TW11_1007	XRE family transcriptional regulator	-0.92	0.0039	**-1.09**	**0.0000**	-0.83	0.0001	**-1.20**	**0.0000**
TW11_1008	sigma-70 family RNA polymerase sigma factor	0.46	0.1740	0.68	0.0001	**1.18**	**0.0000**	**1.32**	**0.0000**
TW11_1009	helix-turn-helix domain-containing protein	0.42	0.2298	0.71	0.0001	**1.14**	**0.0000**	**1.01**	**0.0010**
TW11_1038	ABC transporter permease	-0.94	0.0005	-0.98	0.0000	-0.67	0.0002	**-1.02**	**0.0000**
TW11_1043	transcription antiterminator LicT	-0.26	0.6396	0.13	0.4967	0.20	0.2922	**-1.07**	**0.0000**
TW11_1048	redox-regulated ATPase YchF	-0.71	0.0447	**-1.02**	**0.0000**	-0.89	0.0000	**-1.08**	**0.0001**
TW11_1066	peptidase	**-1.12**	**0.0006**	**-1.03**	**0.0000**	**-1.14**	**0.0000**	-0.78	0.0081
TW11_1076	hypothetical protein	0.00	0.9972	-0.68	0.1565	-0.37	0.4211	**-1.23**	**0.0013**
TW11_1078	ribosome biogenesis GTPase Der	-0.64	0.0867	**-1.01**	**0.0000**	**-1.04**	**0.0000**	-0.48	0.0844
TW11_1081	TetR/AcrR family transcriptional regulator	-0.96	0.1122	-1.53	0.0004	-1.15	0.0041	**-1.45**	**0.0002**
TW11_1084	ACT domain-containing protein	0.81	0.0035	1.00	0.0000	**1.31**	**0.0000**	**1.67**	**0.0000**
TW11_1085	PFL family protein	0.69	0.0116	0.84	0.0000	**1.04**	**0.0000**	**1.28**	**0.0000**
TW11_1098	aminopeptidase P family protein	-0.10	0.8520	**-1.10**	**0.0000**	**-1.74**	**0.0000**	**-1.80**	**0.0000**
TW11_1108	carbon starvation protein A	-1.28	0.0293	-0.80	0.0886	0.98	0.0810	**5.44**	**0.0000**
TW11_1109	transcriptional regulator	-0.67	0.1869	-0.58	0.0796	0.48	0.1048	**2.69**	**0.0000**
TW11_1110	oligosaccharide deacetylase	-0.05	0.9573	0.08	0.7769	0.42	0.0745	**2.20**	**0.0000**
TW11_1111	lipoprotein signal peptidase	-0.44	0.3135	-0.21	0.3638	-0.18	0.4027	**-1.00**	**0.0002**
TW11_1138	DNA-binding response regulator	0.21	0.7219	0.38	0.1058	**1.09**	**0.0000**	**2.18**	**0.0000**
TW11_1142	PTS fructose transporter subunit IIB	0.34	0.4874	0.80	0.0004	**1.07**	**0.0000**	0.88	0.0073
TW11_1143	PTS mannose transporter subunit IIAB	0.24	0.6408	0.70	0.0008	**1.11**	**0.0000**	**1.04**	**0.0002**
TW11_1146	transposase	-0.47	0.4267	0.62	0.0384	**1.13**	**0.0002**	**2.06**	**0.0000**
TW11_1147	2-oxoglutarate translocator	-0.32	0.6288	0.42	0.1445	**1.57**	**0.0000**	**1.16**	**0.0007**
TW11_1161	hypothetical protein	**-1.57**	**0.0001**	**-1.54**	**0.0000**	**-1.11**	**0.0003**	-0.84	0.0146
TW11_1162	endoglucanase	-0.83	0.0059	**-1.01**	**0.0000**	-0.75	0.0000	-0.99	0.0000
TW11_1167	sodium:dicarboxylate symporter	-0.71	0.0479	-0.96	0.0000	**-1.04**	**0.0000**	-0.75	0.0056

TW11_1186	nicotinate-nucleotide adenylyltransferase	0.72	0.0106	**1.11**	**0.0000**	**1.16**	**0.0000**	0.92	0.0001
TW11_1199	transcriptional antiterminator	0.12	0.8535	0.03	0.9268	-0.18	0.5240	**-1.01**	**0.0026**
TW11_1204	MFS transporter	0.18	0.7431	0.57	0.0105	**1.11**	**0.0000**	**1.87**	**0.0000**
TW11_1205	TetR/AcrR family transcriptional regulator	0.15	0.7772	0.25	0.2850	0.91	0.0001	**2.00**	**0.0000**
TW11_1207	cation-translocating P-type ATPase	**2.97**	**0.0000**	**3.45**	**0.0000**	**3.11**	**0.0000**	**3.81**	**0.0000**
TW11_1211	hypothetical protein	**1.26**	**0.0011**	0.93	0.0029	0.74	0.0000	**1.30**	**0.0000**
TW11_1218	mannose-6-phosphate isomerase, class I	-0.15	0.7496	-0.51	0.0044	-0.67	0.0004	**-1.02**	**0.0001**
TW11_1220	PRD domain-containing protein	-1.08	0.0450	**-1.87**	**0.0000**	**-2.09**	**0.0000**	**-1.37**	**0.0000**
TW11_1221	PTS fructose transporter subunit IIA	-0.88	0.2328	**-1.69**	**0.0000**	**-1.92**	**0.0000**	**-1.15**	**0.0001**
TW11_1222	PTS fructose transporter subunit IIB	-0.88	0.2835	**-1.94**	**0.0000**	**-2.45**	**0.0000**	**-1.80**	**0.0000**
TW11_1223	PTS fructose transporter subunit IIC	-0.87	0.3320	**-1.94**	**0.0000**	**-2.59**	**0.0000**	**-2.10**	**0.0000**
TW11_1224	aminopeptidase P family protein	-0.76	0.3055	**-1.51**	**0.0000**	**-1.56**	**0.0000**	**-1.16**	**0.0025**
TW11_1225	putative C-S lyase	-0.65	0.2503	**-1.07**	**0.0000**	**-1.42**	**0.0000**	**-1.07**	**0.0001**
TW11_1231	hypothetical protein	-0.35	0.4472	-0.37	0.0400	**1.06**	**0.0000**	**1.83**	**0.0000**
TW11_1269	ribosome-associated translation inhibitor RaiA	**1.00**	**0.0051**	0.95	0.0001	**1.13**	**0.0000**	**1.99**	**0.0000**
TW11_1309	AcrB/AcrD/AcrF family protein	-0.48	0.3339	**-1.15**	**0.0000**	**-1.09**	**0.0000**	-0.56	0.0957
TW11_1310	efflux RND transporter periplasmic adaptor	-0.44	0.2856	**-1.09**	**0.0000**	**-1.09**	**0.0000**	-0.35	0.2598
TW11_1314	cell wall hydrolase	0.03	0.9657	-0.29	0.0948	0.08	0.6986	**1.70**	**0.0000**
TW11_1320	flavin reductase like domain protein	**1.13**	**0.0002**	**1.16**	**0.0000**	**1.06**	**0.0000**	**1.05**	**0.0000**
TW11_1325	hypothetical protein	0.34	0.6285	**5.31**	**0.0000**	**6.58**	**0.0000**	**5.99**	**0.0000**
TW11_1326	transcriptional regulator	0.22	0.6535	0.73	0.0000	**1.59**	**0.0000**	0.46	0.1175
TW11_1327	protease	0.03	0.9673	0.60	0.0009	**1.65**	**0.0000**	0.44	0.1321
TW11_1328	peptidoglycan-binding protein	0.22	0.6594	0.70	0.0002	**1.78**	**0.0000**	0.89	0.0025
TW11_1333	hypothetical protein	0.20	0.6592	0.31	0.1085	-0.16	0.4903	**-1.12**	**0.0000**
TW11_1334	hypothetical protein	-0.29	0.4975	-0.48	0.0170	**-1.24**	**0.0000**	**-1.06**	**0.0002**
TW11_1376	aspT/YidE/YbjL antiporter duplication domain	-0.28	0.5317	**-1.08**	**0.0000**	-0.32	0.2442	0.58	0.1528
TW11_1378	succinate-semialdehyde dehydrogenase	0.00	0.9968	-0.47	0.0111	0.04	0.8617	**1.14**	**0.0000**
TW11_1379	4-hydroxybutyryl-CoA dehydratase	0.01	0.9919	-0.20	0.3111	0.36	0.0655	**1.05**	**0.0000**
TW11_1380	hypothetical protein	0.00	0.9980	-0.42	0.0650	0.38	0.0652	**1.10**	**0.0000**
TW11_1382	iron-containing alcohol dehydrogenase	0.12	0.8231	-0.09	0.6884	0.45	0.0385	**1.06**	**0.0002**
TW11_1386	PTS mannitol transporter subunit IIB	**-1.12**	**0.0076**	**-1.54**	**0.0000**	**-1.54**	**0.0000**	-0.76	0.0216
TW11_1387	transcription antiterminator BglG	**-1.27**	**0.0029**	**-1.45**	**0.0000**	**-1.50**	**0.0000**	**-1.06**	**0.0055**
TW11_1388	PTS mannose transporter subunit IIA	**-1.03**	**0.0024**	**-1.24**	**0.0000**	**-1.02**	**0.0000**	-0.69	0.0280
TW11_1389	mannitol-1-phosphate 5-dehydrogenase	**-1.09**	**0.0018**	**-1.22**	**0.0000**	**-1.23**	**0.0000**	**-1.02**	**0.0023**
TW11_1390	xanthine phosphoribosyltransferase	-0.35	0.4527	-0.27	0.1354	-0.44	0.0157	**-1.02**	**0.0002**
TW11_1393	PTS fructose transporter subunit IIA	**1.84**	**0.0000**	**1.15**	**0.0002**	-0.55	0.1038	**-1.73**	**0.0000**
TW11_1394	PTS galactitol transporter subunit IIB	**2.16**	**0.0000**	**1.44**	**0.0000**	0.08	0.8132	**-1.33**	**0.0000**
TW11_1395	PTS galactitol transporter subunit IIC	**2.05**	**0.0000**	**1.28**	**0.0000**	-0.33	0.3999	**-2.25**	**0.0000**

TW11_1396	galactitol-1-phosphate 5-dehydrogenase	**2.27**	**0.0000**	**1.21**	**0.0000**	-0.01	0.9792	**-1.14**	**0.0000**
TW11_1397	sorbitol dehydrogenase	**2.22**	**0.0000**	0.93	0.0002	-0.33	0.4315	**-1.11**	**0.0002**
TW11_1398	transketolase	0.43	0.2908	-0.42	0.0124	**-1.02**	**0.0000**	-0.85	0.0014
TW11_1410	cold-shock protein	-1.00	0.0183	-0.82	0.0003	-0.72	0.0170	**-1.98**	**0.0000**
TW11_1412	transcriptional regulator	-0.18	0.7577	-0.04	0.8803	0.35	0.1340	**1.15**	**0.0002**
TW11_1413	hypothetical protein	-0.26	0.5776	-0.11	0.6501	0.35	0.1320	**1.24**	**0.0001**
TW11_1418	membrane protein	0.60	0.0654	0.90	0.0000	0.72	0.0001	**1.09**	**0.0000**
TW11_1421	transcriptional regulator	0.43	0.4426	0.66	0.0246	0.82	0.0060	**1.27**	**0.0007**
TW11_1422	DNA-binding response regulator	0.42	0.2567	0.32	0.0985	0.54	0.0087	**1.24**	**0.0000**
TW11_1429	hypothetical protein	0.42	0.3633	**1.20**	**0.0000**	**1.14**	**0.0000**	**1.08**	**0.0001**
TW11_1437	sodium:alanine symporter family protein	0.10	0.8705	0.99	0.0000	**1.18**	**0.0000**	0.89	0.0016
TW11_1443	1-phosphofructokinase	**-4.20**	**0.0000**	**-4.56**	**0.0000**	**-4.42**	**0.0000**	**-3.43**	**0.0000**
TW11_1444	PTS fructose transporter subunit IIC	**-3.12**	**0.0000**	**-3.31**	**0.0000**	**-3.24**	**0.0000**	**-3.34**	**0.0000**
TW11_1447	membrane protein	**-1.10**	**0.0001**	-0.66	0.0006	-0.44	0.0329	-0.64	0.0223
TW11_1449	peptidylprolyl isomerase	-0.58	0.1020	-0.92	0.0000	-0.94	0.0000	**1.06**	**0.0000**
TW11_1451	aminopeptidase P family protein	-0.36	0.3165	**-1.28**	**0.0000**	**-1.56**	**0.0000**	**-1.50**	**0.0000**
TW11_1465	VWA domain-containing protein	0.03	0.9685	-0.64	0.0004	**-1.06**	**0.0000**	-0.72	0.0342
TW11_1473	putative N-acetylmannosamine-6-phosphate	-1.91	0.0236	**-2.42**	**0.0003**	**-2.87**	**0.0000**	**-1.58**	**0.0000**
TW11_1474	N-acetylneuraminate lyase	-2.26	0.0125	**-2.83**	**0.0003**	**-3.52**	**0.0000**	**-2.48**	**0.0000**
TW11_1475	sodium:solute symporter	**-2.60**	**0.0016**	**-3.58**	**0.0000**	**-4.38**	**0.0000**	**-3.31**	**0.0000**
TW11_1476	YhcH/YjgK/YiaL family protein	**-2.58**	**0.0021**	**-3.27**	**0.0000**	**-3.91**	**0.0000**	**-2.71**	**0.0000**
TW11_1477	hypothetical protein	**-1.96**	**0.0013**	**-2.61**	**0.0000**	**-2.97**	**0.0000**	**-1.51**	**0.0072**
TW11_1478	ROK family protein	**-1.63**	**0.0000**	**-1.68**	**0.0000**	**-1.66**	**0.0000**	-0.70	0.2184
TW11_1479	MurR/RpiR family transcriptional regulator	**-1.17**	**0.0001**	-0.95	0.0000	-0.85	0.0000	-0.47	0.2357
TW11_1480	Crp/Fnr family transcriptional regulator	**-1.34**	**0.0000**	**-1.06**	**0.0000**	-0.93	0.0000	-0.77	0.0323
TW11_1494	carbonic anhydrase	**1.28**	**0.0000**	**1.23**	**0.0000**	**0.53**	0.0037	0.77	0.0037
TW11_1495	MarR family transcriptional regulator	-0.82	0.0069	**-1.07**	**0.0000**	-0.74	0.0001	**1.37**	**0.0000**
TW11_1496	ABC transporter ATP-binding protein	-0.84	0.0059	**-1.31**	**0.0000**	**-1.35**	**0.0000**	0.66	0.0193
TW11_1497	ABC transporter ATP-binding protein	-0.60	0.0720	**-1.00**	**0.0000**	**-1.15**	**0.0000**	0.69	0.0081
TW11_1499	MarR family transcriptional regulator	0.10	0.8935	0.39	0.0346	0.75	0.0001	**1.24**	**0.0000**
TW11_1503	esterase family protein	0.77	0.0070	**1.17**	**0.0000**	0.79	0.0000	0.60	0.0193
TW11_1515	cell surface protein	-0.38	0.3156	-0.23	0.2219	**1.01**	**0.0000**	**1.99**	**0.0000**
TW11_1516	membrane protein	-0.16	0.7666	-0.03	0.9032	**1.15**	**0.0000**	**1.38**	**0.0000**
TW11_1517	PHP domain-containing protein	0.25	0.6128	0.45	0.0546	0.97	0.0000	**2.25**	**0.0000**
TW11_1540	4Fe-4S ferredoxin	**-1.06**	**0.0000**	**-1.76**	**0.0000**	-0.87	0.0000	-0.34	0.2283
TW11_1541	hydroxylamine reductase	**-1.07**	**0.0000**	**-1.96**	**0.0000**	**-1.65**	**0.0000**	**-1.18**	**0.0000**
TW11_1559	hypothetical protein	0.65	0.0836	0.54	0.0120	0.84	0.0002	**2.05**	**0.0000**
TW11_1565	sporulation membrane protein YtaF	-0.71	0.1270	-0.55	0.0660	-0.19	0.5338	**1.14**	**0.0017**

TW11_1589	1-deoxy-D-xylulose-5-phosphate reductoisomerase	-0.41	0.2208	-0.87	0.0000	-1.01	0.0000	**-0.56**	**0.0169**
TW11_1606	translational GTPase TypA	**-1.30**	**0.0000**	**-1.38**	**0.0000**	**-1.18**	**0.0000**	-0.83	0.0019
TW11_1610	DNA-binding response regulator	-0.39	0.2503	-0.85	0.0000	**-1.01**	**0.0000**	0.19	0.5007
TW11_1611	two-component sensor histidine kinase	-0.44	0.1706	-0.94	0.0000	**-1.12**	**0.0000**	0.15	0.5940
TW11_1617	NCS2 family permease	-0.17	0.8030	**1.32**	**0.0000**	**2.19**	**0.0000**	**2.00**	**0.0000**
TW11_1619	ABC transporter ATP-binding protein	-0.41	0.4406	0.20	0.5269	0.60	0.0146	**1.64**	**0.0000**
TW11_1625	(2Fe-2S)-binding protein	-0.38	0.3320	-0.82	0.0000	**-1.14**	**0.0000**	-0.76	0.0047
TW11_1660	transcriptional regulator	0.21	0.6842	0.66	0.0007	**1.05**	**0.0000**	-0.19	0.5823
TW11_1661	transcriptional regulator	0.48	0.2501	**1.18**	**0.0000**	**2.07**	**0.0000**	**3.17**	**0.0000**
TW11_1662	hypothetical protein	0.54	0.1773	**1.36**	**0.0000**	**1.18**	**0.0000**	0.78	0.0036
TW11_1663	hypothetical protein	0.65	0.0492	**1.34**	**0.0000**	**1.19**	**0.0000**	0.97	0.0001
TW11_1666	glutamine--tRNA ligase	0.23	0.5970	-0.41	0.0108	**-1.01**	**0.0000**	-0.85	0.0003
TW11_1679	transcriptional regulator	0.59	0.1149	**3.20**	**0.0000**	**4.49**	**0.0000**	**4.11**	**0.0000**
TW11_1680	hypothetical protein	0.43	0.2793	**3.12**	**0.0000**	**4.39**	**0.0000**	**4.42**	**0.0000**
TW11_1685	hypothetical protein	0.76	0.0590	**1.26**	**0.0000**	**1.22**	**0.0000**	**1.19**	**0.0000**
TW11_1697	GntR family transcriptional regulator	0.46	0.3320	0.47	0.0544	0.74	0.0021	**1.32**	**0.0001**
TW11_1698	plasmid replication protein	**1.28**	**0.0000**	**1.64**	**0.0000**	**1.75**	**0.0000**	**1.73**	**0.0000**
TW11_1699	hypothetical protein	**1.21**	**0.0001**	**1.76**	**0.0000**	**1.77**	**0.0000**	**1.26**	**0.0000**
TW11_1700	ATP-dependent chaperone ClpB	0.72	0.0781	0.73	0.0000	0.90	0.0000	**1.07**	**0.0001**
TW11_1702	AraC family transcriptional regulator	0.26	0.6772	0.97	0.0000	**1.25**	**0.0000**	0.73	0.0099
TW11_1706	VanZ family protein	0.33	0.6264	0.82	0.0115	0.76	0.0235	**1.41**	**0.0001**
TW11_1709	hypothetical protein	0.22	0.6782	0.10	0.7465	0.66	0.0056	**1.18**	**0.0001**
TW11_1710	transposase	0.30	0.5043	0.29	0.2185	0.91	0.0000	**1.14**	**0.0001**
TW11_1711	ATP-binding protein	0.09	0.8832	0.23	0.2666	0.76	0.0000	**1.34**	**0.0000**
TW11_1712	XRE family transcriptional regulator	0.29	0.4858	0.38	0.0668	**1.03**	**0.0000**	**1.45**	**0.0000**
TW11_1713	conjugal transfer protein	0.38	0.4899	0.39	0.2182	**1.15**	**0.0000**	**1.46**	**0.0000**
TW11_1714	antirestriction protein ArdA	0.22	0.7143	0.38	0.1445	0.90	0.0002	**1.53**	**0.0000**
TW11_1715	ATP/GTP-binding protein	0.16	0.7457	0.17	0.4591	0.95	0.0000	**1.41**	**0.0000**
TW11_1716	membrane protein	0.24	0.6123	0.27	0.1970	**1.15**	**0.0000**	**1.87**	**0.0000**
TW11_1717	peptidase P60	0.09	0.8987	0.17	0.5292	**1.11**	**0.0000**	**1.72**	**0.0000**
TW11_1718	conjugal transfer protein	0.27	0.5776	0.35	0.1343	**1.21**	**0.0000**	**1.70**	**0.0000**
TW11_1720	XRE family transcriptional regulator	-0.95	0.0031	**-1.08**	**0.0000**	-0.77	0.0004	**-1.23**	**0.0000**
TW11_1721	sigma-70 family RNA polymerase sigma factor	0.48	0.1274	0.71	0.0001	**1.22**	**0.0000**	**1.36**	**0.0000**
TW11_1722	helix-turn-helix domain-containing protein	0.44	0.1865	0.70	0.0001	**1.14**	**0.0000**	0.98	0.0012
TW11_1734	membrane protein	**-1.25**	**0.0017**	-0.78	0.0086	-0.86	0.0047	-0.39	0.4239
TW11_1750	AraC family transcriptional regulator	0.56	0.1517	0.89	0.0000	**1.08**	**0.0000**	-0.23	0.4828
TW11_1768	propanediol utilization protein PduU	0.68	0.0479	**1.09**	**0.0000**	**1.22**	**0.0000**	0.34	0.3191
TW11_1781	XRE family transcriptional regulator	**1.10**	**0.0000**	0.92	0.0000	0.60	0.0015	0.06	0.8624

TW11_1782	hypothetical protein	**1.23**	**0.0000**	0.96	0.0000	0.42	0.0631	0.05	0.8995
TW11_1783	hypothetical protein	**1.18**	**0.0000**	0.90	0.0000	0.30	0.1976	0.10	0.7897
TW11_1784	outer dense fiber protein 1	**1.31**	**0.0000**	**1.04**	**0.0000**	0.36	0.1090	0.09	0.8149
TW11_1785	DNA polymerase IV	**1.01**	**0.0006**	0.69	0.0004	0.14	0.5433	-0.02	0.9636
TW11_1796	endonuclease	0.89	0.0017	**1.17**	**0.0000**	**1.69**	**0.0000**	1.00	0.0001
TW11_1804	group II intron reverse transcriptase/maturase	0.01	0.9848	0.41	0.0814	**1.04**	**0.0000**	**1.13**	**0.0001**
TW11_1805	conjugal transfer protein	-0.16	0.8935	0.97	0.0106	**1.36**	**0.0001**	**2.08**	**0.0000**
TW11_1806	hypothetical protein	0.26	0.5957	0.26	0.2626	**1.04**	**0.0000**	-0.11	0.7571
TW11_1817	Lsa family ABC-F type ribosomal protection	0.30	0.4792	0.63	0.0002	**1.13**	**0.0000**	0.91	0.0001
TW11_1818	helicase	0.25	0.7230	0.55	0.0843	**1.11**	**0.0004**	0.05	0.9153
TW11_1855	hypothetical protein	**1.15**	**0.0000**	**1.20**	**0.0000**	0.82	0.0000	0.75	0.0064
TW11_1856	YtxH domain-containing protein	**1.23**	**0.0000**	**1.21**	**0.0000**	0.99	0.0000	0.92	0.0009
TW11_1877	ATP-dependent zinc metalloprotease FtsH	0.62	0.0339	0.75	0.0000	0.55	0.0007	**1.47**	**0.0000**
TW11_1878	MarR family transcriptional regulator	0.85	0.0081	**1.30**	**0.0000**	**1.55**	**0.0000**	**3.11**	**0.0000**
TW11_1892	LytTR family transcriptional regulator	0.30	0.5630	0.90	0.0000	**1.30**	**0.0000**	0.27	0.4338
TW11_1893	histidine kinase	0.23	0.6573	0.59	0.0113	**1.20**	**0.0000**	0.09	0.8325
TW11_1907	LysR family transcriptional regulator	0.93	0.0033	**1.24**	**0.0000**	**1.13**	**0.0000**	0.95	0.0003
TW11_1912	oxidoreductase	-0.24	0.7285	-0.97	0.0000	**-1.55**	**0.0000**	**-1.77**	**0.0000**
TW11_1938	membrane protein	-0.31	0.5215	-0.99	0.0000	**-1.31**	**0.0000**	**-1.33**	**0.0000**
TW11_1939	type I glyceraldehyde-3-phosphate dehydrogenase	-0.21	0.6175	-0.18	0.3789	0.33	0.1010	**2.34**	**0.0000**
TW11_1949	pyrimidine dimer DNA glycosylase	0.03	0.9681	0.15	0.5492	0.50	0.0249	**1.53**	**0.0000**
TW11_1951	transcriptional regulator	-0.09	0.9106	-0.01	0.9786	0.35	0.1944	**1.81**	**0.0000**
TW11_1952	ABC transporter permease	-0.40	0.4033	-0.09	0.7815	0.09	0.7726	**1.73**	**0.0000**
TW11_1953	ABC transporter permease	-0.53	0.1938	-0.56	0.0177	-0.03	0.9223	**1.79**	**0.0000**
TW11_1954	ABC transporter ATP-binding protein	-0.43	0.2786	-0.52	0.0172	0.02	0.9541	**2.12**	**0.0000**
TW11_1955	TetR/AcrR family transcriptional regulator	-0.19	0.7093	-0.27	0.1350	-0.17	0.4520	**1.06**	**0.0000**
TW11_1965	hypothetical protein	-0.21	0.7061	-0.09	0.7410	0.22	0.3690	**1.16**	**0.0001**
TW11_1990	AraC family transcriptional regulator	**1.22**	**0.0027**	**1.11**	**0.0004**	0.91	0.0006	1.00	0.0144
TW11_1991	Na/Pi cotransporter family protein	**-1.33**	**0.0000**	**-1.31**	**0.0000**	**-1.10**	**0.0000**	-0.30	0.4755
TW11_2001	hydantoinase	0.68	0.0202	0.79	0.0000	**1.15**	**0.0000**	**1.17**	**0.0000**
TW11_2002	hypothetical protein	0.74	0.0234	0.69	0.0001	**1.07**	**0.0000**	**1.29**	**0.0000**
TW11_2003	cytosine permease	0.81	0.0034	0.83	0.0000	**1.24**	**0.0000**	**1.28**	**0.0000**
TW11_2020	bifunctional	0.99	0.0021	0.86	0.0077	0.18	0.5195	**1.37**	**0.0001**
TW11_2021	riboflavin synthase	**1.04**	**0.0039**	0.73	0.0534	-0.01	0.9728	**1.51**	**0.0002**
TW11_2022	bifunctional	0.98	0.0214	0.71	0.0561	0.14	0.6878	**1.80**	**0.0000**
TW11_2023	6,7-dimethyl-8-ribityllumazine synthase	0.95	0.0122	0.79	0.0090	0.34	0.2191	**1.57**	**0.0000**
TW11_2024	hypothetical protein	0.48	0.3453	0.91	0.0000	**1.07**	**0.0000**	0.34	0.2331
TW11_2027	hypothetical protein	0.83	0.0034	**1.05**	**0.0000**	0.90	0.0000	0.59	0.0195

TW11_2037	radical SAM protein	-0.19	0.6687	-0.77	0.0000	-1.01	0.0000	-0.88	0.0002
TW11_2038	membrane protein	-0.25	0.5542	-0.82	0.0000	-1.09	0.0000	-1.06	0.0000
TW11_2039	nucleoside hydrolase	-0.28	0.4782	-0.93	0.0000	-1.10	0.0000	-0.96	0.0000
TW11_2054	hypothetical protein	0.45	0.2967	0.69	0.0003	1.16	0.0000	0.73	0.0071
TW11_2055	hypothetical protein	0.96	0.2677	1.72	0.0006	1.61	0.0024	1.51	0.0363
TW11_2056	hypothetical protein	0.01	0.9948	0.70	0.1014	0.76	0.1019	1.58	0.0018
TW11_2061	guanine deaminase	-0.39	0.3123	-1.22	0.0000	-1.29	0.0000	-1.10	0.0000
TW11_2063	guanine deaminase	-0.33	0.4584	-0.58	0.0035	-0.46	0.0329	-1.05	0.0001
TW11_2064	hypothetical protein	0.05	0.9478	-0.52	0.0056	-0.87	0.0000	-1.62	0.0000
TW11_2066	glycine dehydrogenase subunit 2	0.94	0.0002	1.01	0.0000	1.25	0.0000	0.84	0.0002
TW11_2067	aminomethyl-transferring glycine dehydrogenase	0.93	0.0005	0.86	0.0000	1.09	0.0000	0.96	0.0003
TW11_2070	lipoate--protein ligase A	0.09	0.9090	-0.63	0.0007	-0.62	0.0059	-1.04	0.0028
TW11_2078	peptidase M19	0.28	0.5063	0.45	0.0100	0.71	0.0001	1.02	0.0001
TW11_2099	MFS transporter	-0.81	0.0255	-0.47	0.0362	0.00	0.9962	-1.09	0.0002
TW11_2121	GIY-YIG nuclease family protein	1.10	0.0534	0.91	0.0396	1.52	0.0002	1.36	0.0111
TW11_2122	zinc metalloprotease Zmp1	0.02	0.9864	-0.05	0.8795	0.56	0.0272	1.20	0.0001
TW11_2128	potassium-transporting ATPase subunit B	-0.91	0.0092	-1.05	0.0000	-0.77	0.0002	-1.03	0.0001
TW11_2129	potassium-transporting ATPase subunit KdpA	-1.09	0.0001	-0.99	0.0000	-0.87	0.0000	-1.08	0.0004
TW11_2132	N-acetylglucosamine kinase	1.12	0.0246	0.91	0.0186	1.01	0.0093	0.58	0.2877
TW11_2157	hypothetical protein	-0.46	0.2559	-1.05	0.0000	-0.68	0.0025	-0.13	0.7052
TW11_2161	polysaccharide deacetylase	0.01	0.9858	-0.46	0.0181	-0.21	0.3214	1.03	0.0001
TW11_2162	amino acid permease	-1.02	0.0010	-0.92	0.0000	-0.65	0.0066	-0.51	0.1321
TW11_2172	channel protein, hemolysin III family	-0.37	0.3165	-0.86	0.0001	-1.24	0.0000	-1.38	0.0000
TW11_2173	hypothetical protein	-0.34	0.3849	-0.91	0.0000	-1.40	0.0000	-1.46	0.0000
TW11_2200	GNAT family N-acetyltransferase	1.18	0.0000	0.99	0.0000	0.49	0.0198	0.48	0.1047
TW11_2205	hypothetical protein	-0.48	0.2151	-0.38	0.0548	0.29	0.1906	1.11	0.0000
TW11_2206	3-methyl-2-oxobutanoate	-0.30	0.5043	-0.53	0.0046	0.27	0.1923	1.02	0.0000
TW11_2207	pantoate--beta-alanine ligase	-0.19	0.7223	-0.39	0.0785	0.30	0.1755	1.02	0.0001
TW11_2208	hypothetical protein	0.78	0.0117	1.14	0.0000	1.29	0.0000	-0.15	0.6516
TW11_2211	4Fe-4S binding protein	0.47	0.3535	0.52	0.0746	0.74	0.0034	1.04	0.0009
TW11_2216	ABC transporter ATP-binding protein	-0.04	0.9531	-0.53	0.0049	-1.05	0.0000	-0.31	0.3222
TW11_2240	hypothetical protein	1.39	0.0002	0.64	0.0609	0.13	0.5253	1.24	0.0003
TW11_2245	FeoB-associated Cys-rich membrane protein	1.80	0.0000	1.46	0.0000	1.27	0.0000	1.64	0.0000
TW11_2246	ferrous iron transport protein B	1.48	0.0000	0.98	0.0046	0.79	0.0021	1.51	0.0000
TW11_2247	iron transporter FeoA	1.28	0.0008	1.11	0.0002	1.06	0.0003	1.65	0.0000
TW11_2248	ferrous iron transport protein A	1.09	0.0082	0.73	0.0215	0.72	0.0009	1.77	0.0000
TW11_2314	D-alanine--D-alanine ligase	-0.88	0.0041	-1.67	0.0000	-2.80	0.0000	-3.11	0.0000
TW11_2315	PLP-dependent aminotransferase family protein	-0.90	0.0013	-1.10	0.0000	-1.56	0.0000	-1.68	0.0000

TW11_2336	hypothetical protein	-0.22	0.7431	0.24	0.4485	0.39	0.1710	**1.09**	**0.0005**
TW11_2339	allophanate hydrolase	0.02	0.9775	0.65	0.0014	**1.88**	**0.0000**	**2.65**	**0.0000**
TW11_2340	allophanate hydrolase subunit 1	-0.42	0.4214	0.30	0.2136	**1.32**	**0.0000**	**2.43**	**0.0000**
TW11_2341	divalent metal cation transporter	-0.56	0.3078	-0.05	0.8650	0.95	0.0001	**2.15**	**0.0000**
TW11_2342	LamB/YcsF family protein	-0.81	0.0358	-0.33	0.1501	0.29	0.2326	**1.27**	**0.0000**
TW11_2345	TetR/AcrR family transcriptional regulator	-0.08	0.9288	-0.19	0.5538	-0.19	0.5395	**1.01**	**0.0014**
TW11_2363	phage portal protein	0.29	0.5469	0.82	0.0000	**1.45**	**0.0000**	**1.48**	**0.0000**
TW11_2364	phage portal protein	0.28	0.5628	0.84	0.0000	**1.33**	**0.0000**	**1.51**	**0.0000**
TW11_2365	phage portal protein	0.34	0.4097	**1.06**	**0.0000**	**1.43**	**0.0000**	**1.31**	**0.0000**
TW11_2366	hypothetical protein	**1.65**	**0.0000**	**4.21**	**0.0000**	**5.32**	**0.0000**	**3.49**	**0.0000**
TW11_2369	XRE family transcriptional regulator	0.99	0.0005	**1.12**	**0.0000**	**1.46**	**0.0000**	**1.53**	**0.0000**
TW11_2370	ImmA/IrrE family metallo-endopeptidase	0.66	0.0365	0.72	0.0001	**1.06**	**0.0000**	0.44	0.1098
TW11_2385	galactosyldiacylglycerol synthase	0.67	0.0451	**1.22**	**0.0000**	**1.03**	**0.0000**	0.72	0.0048
TW11_2398	PAS domain S-box protein	-0.76	0.0045	-0.95	0.0000	-0.65	0.0002	**-1.36**	**0.0000**
TW11_2410	polyribonucleotide nucleotidyltransferase	-0.27	0.6426	-0.95	0.0000	**-1.12**	**0.0000**	-0.57	0.0610
TW11_2426	hypothetical protein	0.22	0.7431	0.71	0.0100	**1.04**	**0.0003**	0.88	0.0229
TW11_2437	D-alanyl-D-alanine carboxypeptidase	**-1.11**	**0.0000**	**-1.29**	**0.0000**	**-1.22**	**0.0000**	**-1.33**	**0.0004**
TW11_2438	spore protein	**-1.37**	**0.0001**	**-1.44**	**0.0000**	-0.92	0.0005	**-1.23**	**0.0031**
TW11_2439	ribonuclease J	-0.56	0.0534	-0.91	0.0000	-0.93	0.0000	**-1.38**	**0.0000**
TW11_2440	metal-dependent hydrolase	-0.54	0.0871	-0.89	0.0000	**-1.12**	**0.0000**	**-1.60**	**0.0000**
TW11_2450	Rrf2 family transcriptional regulator	-0.05	0.9400	-0.58	0.0006	**-1.28**	**0.0000**	-0.91	0.0004
TW11_2454	type I DNA topoisomerase	-0.58	0.0798	-0.81	0.0000	**-1.03**	**0.0000**	-0.41	0.1504
TW11_2469	branched-chain amino acid transport system II	-0.55	0.1030	-0.51	0.0087	-0.38	0.0818	**-1.04**	**0.0000**
TW11_2470	ribosome biogenesis GTPase YlqF	**-1.13**	**0.0001**	-0.76	0.0002	-0.72	0.0008	-0.96	0.0006
TW11_2477	putative DNA-binding protein	-0.13	0.8186	0.00	0.9894	0.34	0.1290	**1.05**	**0.0001**
TW11_2494	hypothetical protein	0.15	0.7897	0.73	0.0005	**1.09**	**0.0000**	0.72	0.0213
TW11_2501	penicillin-binding protein 2	**-2.00**	**0.0000**	**-2.10**	**0.0000**	**-1.80**	**0.0000**	**-2.02**	**0.0000**
TW11_2502	peptidase U32	**-1.04**	**0.0000**	**-1.21**	**0.0000**	**-1.02**	**0.0000**	**-1.15**	**0.0000**
TW11_2503	O-methyltransferase	**-1.21**	**0.0000**	**-1.07**	**0.0000**	-0.76	0.0000	-0.86	0.0002
TW11_2530	Asp23/Gls24 family envelope stress response	0.97	0.0028	0.93	0.0000	**1.06**	**0.0000**	**1.78**	**0.0000**
TW11_2561	hypothetical protein	-0.80	0.0123	**-1.44**	**0.0000**	**-1.09**	**0.0000**	-0.87	0.0015
TW11_2576	hypothetical protein	-0.08	0.9227	0.00	0.9944	0.50	0.0186	**1.37**	**0.0000**
TW11_2577	PadR family transcriptional regulator	-0.10	0.9114	0.12	0.7442	0.82	0.0014	**1.88**	**0.0000**
TW11_2611	AraC family transcriptional regulator	**-1.26**	**0.0000**	-0.42	0.0424	-0.07	0.7714	-0.85	0.0009
TW11_2623	zinc transporter ZupT	**1.42**	**0.0000**	**1.02**	**0.0002**	**1.01**	**0.0000**	**1.92**	**0.0000**
TW11_2631	LysR family transcriptional regulator	0.80	0.0103	**1.14**	**0.0000**	**1.24**	**0.0000**	0.22	0.4802
TW11_2633	PTS sugar transporter subunit IIC	-0.85	0.0238	**-1.28**	**0.0000**	-0.78	0.0006	-0.54	0.0499
TW11_2634	PTS mannose/fructose/sorbose transporter subunit	-0.87	0.0260	**-1.39**	**0.0000**	**-1.06**	**0.0000**	-0.63	0.0346

TW11_2635	membrane complex biogenesis protein, BtpA	-0.99	0.0016	**-1.47**	**0.0000**	**-1.34**	**0.0000**	-0.81	0.0033
TW11_2636	PTS mannose transporter subunit IIC	**-1.14**	**0.0000**	**-1.24**	**0.0000**	**-1.12**	**0.0000**	-0.88	0.0018
TW11_2637	GntR family transcriptional regulator	**-1.11**	**0.0000**	**-1.09**	**0.0000**	**-1.13**	**0.0000**	-0.89	0.0012
TW11_2647	hypothetical protein	-1.36	0.1420	**-1.41**	**0.0018**	**-1.55**	**0.0006**	**-2.12**	**0.0000**
TW11_2650	hypothetical protein	**-1.30**	**0.0029**	-0.35	0.3026	-0.07	0.8766	-0.43	0.3343
TW11_2651	acyl carrier protein	0.65	0.0355	0.60	0.0007	0.11	0.6888	**-1.17**	**0.0000**
TW11_2658	electron transfer flavoprotein subunit beta/FixA	-0.41	0.3304	**-1.06**	**0.0000**	-0.41	0.0679	-0.24	0.3852
TW11_2669	HlyC/CorC family transporter	0.63	0.0675	**1.00**	**0.0000**	**1.24**	**0.0000**	0.78	0.0010
TW11_2693	spermidine/putrescine ABC transporter	0.26	0.5404	0.39	0.0348	0.53	0.0227	**1.27**	**0.0000**
TW11_2694	transcriptional regulator	0.66	0.1134	0.72	0.0009	**1.00**	**0.0004**	**1.95**	**0.0000**
TW11_2695	membrane protein	0.14	0.8181	0.84	0.0000	**1.29**	**0.0000**	**1.02**	**0.0006**
TW11_2703	GGDEF domain-containing protein	0.63	0.1001	**1.34**	**0.0000**	**1.73**	**0.0000**	0.01	0.9852
TW11_2707	glucosamine-6-phosphate deaminase	**-2.57**	**0.0000**	**-3.46**	**0.0000**	**-4.24**	**0.0000**	**-3.47**	**0.0000**
TW11_2708	N-acetylglucosamine-6-phosphate deacetylase	**-2.83**	**0.0001**	**-4.21**	**0.0000**	**-4.73**	**0.0000**	**-3.53**	**0.0000**
TW11_2709	GntR family transcriptional regulator	**-2.97**	**0.0000**	**-4.12**	**0.0000**	**-4.69**	**0.0000**	**-3.05**	**0.0000**
TW11_2710	DNA-binding protein	-0.43	0.2664	-0.42	0.0167	-0.42	0.0305	**-1.03**	**0.0000**
TW11_2715	transcriptional regulator	0.96	0.0003	**1.21**	**0.0000**	0.92	0.0000	0.82	0.0011
TW11_2721	ABC transporter ATP-binding protein	-0.98	0.0014	**-1.07**	**0.0000**	**-1.25**	**0.0000**	**-1.01**	**0.0001**
TW11_2722	ABC transporter permease	-1.00	0.0023	**-1.16**	**0.0000**	**-1.11**	**0.0000**	**-1.10**	**0.0002**
TW11_2726	hypothetical protein	**-1.85**	**0.0001**	-0.85	0.0199	**-1.04**	**0.0053**	**-1.34**	**0.0034**
TW11_2727	hypothetical protein	1.12	0.0129	0.68	0.0017	0.48	0.0841	**1.59**	**0.0000**
TW11_2728	3-phosphoglycerate dehydrogenase	1.18	0.0356	0.41	0.1026	0.27	0.4308	**1.66**	**0.0000**
TW11_2729	alanine--glyoxylate aminotransferase family	**1.17**	**0.0059**	0.62	0.0047	0.53	0.0454	**1.68**	**0.0000**
TW11_2743	aldo/keto reductase	0.86	0.0074	**1.12**	**0.0000**	**1.11**	**0.0000**	0.73	0.0038
TW11_2744	cation transporter	**-1.34**	**0.0005**	**-1.07**	**0.0001**	-0.74	0.0132	**-1.08**	**0.0085**
TW11_2745	ABC transporter permease	-0.49	0.2462	**-1.10**	**0.0000**	-0.93	0.0000	-0.79	0.0014
TW11_2746	proline/glycine betaine ABC transporter	-0.67	0.0369	**-1.09**	**0.0000**	**-1.04**	**0.0000**	-0.85	0.0011
TW11_2751	MBL fold protein	-0.79	0.0100	**-1.17**	**0.0000**	**-1.16**	**0.0000**	**-1.24**	**0.0000**
TW11_2752	iron hydrogenase	-0.56	0.0655	-0.93	0.0000	-0.94	0.0000	**-1.17**	**0.0000**
TW11_2754	cold-shock protein	-0.15	0.7841	0.09	0.7318	0.16	0.6285	**-1.08**	**0.0000**
TW11_2770	ABC transporter	0.25	0.6413	0.55	0.0240	0.89	0.0001	**2.40**	**0.0000**
TW11_2771	ABC transporter substrate-binding protein	0.14	0.8097	0.71	0.0016	0.91	0.0000	**1.96**	**0.0000**
TW11_2778	molybdate ABC transporter substrate-binding	-0.73	0.0204	-0.83	0.0000	-0.35	0.1140	**1.13**	**0.0000**
TW11_2789	ABC transporter ATP-binding protein	0.73	0.0116	0.82	0.0000	**1.18**	**0.0000**	0.88	0.0003
TW11_2803	hypothetical protein	0.11	0.8878	0.25	0.3928	**1.07**	**0.0000**	0.40	0.3752
TW11_2805	nuclease	-0.69	0.0454	**-1.16**	**0.0000**	**-1.35**	**0.0000**	**-1.22**	**0.0003**
TW11_2806	cell wall-binding protein Cwp25	0.94	0.0003	**1.15**	**0.0000**	0.91	0.0000	0.54	0.0366
TW11_2815	iron dependent repressor DNA-binding	0.16	0.7666	0.01	0.9730	0.17	0.4543	**1.13**	**0.0000**

TW11_2816	isocitrate dehydrogenase	0.22	0.6288	0.77	0.0000	**1.63**	**0.0000**	**2.66**	**0.0000**
TW11_2817	aconitate hydratase	0.10	0.8889	0.50	0.0043	**1.33**	**0.0000**	**2.60**	**0.0000**
TW11_2818	homocitrate synthase	0.23	0.7230	**1.33**	**0.0000**	**2.40**	**0.0000**	**3.81**	**0.0000**
TW11_2820	hypothetical protein	0.67	0.1161	0.98	0.0002	**1.30**	**0.0000**	0.44	0.2210
TW11_2821	membrane protein	0.50	0.3309	0.92	0.0012	**1.32**	**0.0000**	0.61	0.1116
TW11_2822	MBL fold metallo-hydrolase	**-1.16**	**0.0000**	-0.97	0.0000	**-1.32**	**0.0000**	**-1.43**	**0.0000**
TW11_2823	transposase	**-1.08**	**0.0002**	-0.87	0.0001	**-1.35**	**0.0000**	**-1.52**	**0.0000**
TW11_2824	MBL fold metallo-hydrolase	-0.94	0.0123	**-1.19**	**0.0000**	**-1.76**	**0.0000**	**-1.93**	**0.0000**
TW11_2825	glutamate synthase	-0.37	0.5449	-0.74	0.0280	**-1.19**	**0.0000**	-0.83	0.0087
TW11_2826	rubredoxin oxidoreductase (desulfoferrodoxin)	-0.42	0.2328	-0.95	0.0006	**-1.29**	**0.0000**	-0.89	0.0009
TW11_2827	transcriptional repressor	-0.51	0.1130	**-1.08**	**0.0001**	**-1.42**	**0.0000**	**-1.04**	**0.0001**
TW11_2828	rubrerythrin	-0.49	0.1164	-0.96	0.0001	**-1.20**	**0.0000**	-0.81	0.0013
TW11_2851	crotonase	-0.12	0.8961	0.15	0.7227	0.24	0.4664	**-1.03**	**0.0013**
TW11_2855	sulfite exporter TauE/SafE family protein	-0.82	0.0905	-0.66	0.0463	-0.40	0.2375	**-1.15**	**0.0020**
TW11_2871	LacI family transcriptional regulator	0.92	0.0022	0.93	0.0000	**1.20**	**0.0000**	0.95	0.0001
TW11_2872	stage V sporulation protein AE	-0.82	0.1420	-0.36	0.3323	-0.42	0.2307	**-1.03**	**0.0057**
TW11_2880	PTS sorbose transporter subunit IIB	**1.35**	**0.0001**	**1.04**	**0.0000**	0.90	0.0003	0.81	0.0185
TW11_2881	PTS sorbitol transporter subunit IIB	**1.30**	**0.0000**	0.65	0.0074	0.70	0.0015	0.30	0.3726
TW11_2882	PTS sorbose transporter subunit IIB	**1.18**	**0.0006**	0.88	0.0003	0.53	0.0273	0.13	0.7173
TW11_2883	PTS sorbitol transporter subunit IIC	**1.19**	**0.0000**	0.67	0.0013	0.42	0.0657	0.37	0.2403
TW11_2884	sorbitol operon activator protein (glucitol)	**1.03**	**0.0021**	0.32	0.2166	0.23	0.4296	0.21	0.5599
TW11_2886	helicase	**-1.12**	**0.0002**	**-1.47**	**0.0000**	**-1.48**	**0.0000**	**-1.27**	**0.0000**
TW11_2889	pyruvate formate lyase-activating protein	-0.97	0.0002	-0.80	0.0000	-0.87	0.0000	**-1.39**	**0.0000**
TW11_2911	zinc transporter	0.82	0.0086	**1.08**	**0.0000**	0.86	0.0000	0.61	0.0166
TW11_2912	hypothetical protein	0.83	0.0022	**1.01**	**0.0000**	0.80	0.0000	0.60	0.0113
TW11_2942	aminoacyl-histidine dipeptidase	0.06	0.9334	-0.60	0.0008	-0.70	0.0003	**-1.75**	**0.0000**
TW11_2985	hypothetical protein	0.05	0.9477	0.11	0.5925	-0.39	0.0576	**-1.00**	**0.0000**
TW11_2988	transcriptional regulator	0.23	0.6848	0.65	0.0011	**1.15**	**0.0000**	0.37	0.2793
TW11_2991	protein utxA	-1.06	0.0108	-0.93	0.0018	-0.74	0.0257	**-1.12**	**0.0013**
TW11_2995	sodium:proton antiporter	0.05	0.9477	-0.10	0.6179	-0.06	0.8509	**-1.32**	**0.0000**
TW11_2996	voltage-gated chloride channel protein	-0.73	0.0423	-0.59	0.0042	-0.24	0.2889	**-1.09**	**0.0001**
TW11_2997	MBL fold metallo-hydrolase	0.63	0.0781	0.98	0.0000	**1.16**	**0.0000**	**1.12**	**0.0000**
TW11_3006	membrane protein	0.37	0.2755	0.47	0.0027	0.66	0.0004	**-1.13**	**0.0000**
TW11_3007	transcriptional regulator	0.16	0.7694	**1.25**	**0.0000**	**1.57**	**0.0000**	**1.07**	**0.0001**
TW11_3008	membrane protein	0.05	0.9490	**1.38**	**0.0000**	**1.71**	**0.0000**	**1.16**	**0.0000**
TW11_3009	hypothetical protein	0.31	0.4584	0.55	0.0035	**1.12**	**0.0000**	0.17	0.5944
TW11_3016	MATE family efflux transporter	-0.03	0.9781	0.13	0.7295	0.41	0.2253	**1.22**	**0.0003**
TW11_3018	4Fe-4S binding protein	-0.89	0.0208	**-1.40**	**0.0000**	**-1.90**	**0.0000**	**-2.02**	**0.0000**

TW11_3019	ferredoxin	-0.32	0.6443	-1.16	0.0005	-1.22	0.0005	-0.73	0.1293
TW11_3020	transcriptional regulator	0.13	0.9010	-0.01	0.9997	-0.27	0.5646	-1.42	0.0001
TW11_3023	transcriptional regulator	-0.65	0.3983	-0.71	0.1405	-1.09	0.0172	-1.27	0.0011
TW11_3032	GrpB family protein	0.68	0.0619	0.92	0.0000	0.85	0.0000	1.29	0.0000
TW11_3050	hypothetical protein	0.88	0.0024	1.06	0.0000	0.83	0.0001	0.56	0.0956
TW11_3051	GGDEF domain-containing protein	0.12	0.8124	0.35	0.0654	1.00	0.0000	0.94	0.0001
TW11_3056	NADP-dependent glyceraldehyde-3-phosphate	-0.46	0.2503	-0.59	0.0009	-1.04	0.0000	-0.53	0.1430
TW11_3057	TetR/AcrR family transcriptional regulator	-1.05	0.0021	-1.46	0.0000	-2.33	0.0000	-1.36	0.0000
TW11_3058	hypothetical protein	-1.11	0.0003	-1.68	0.0000	-2.58	0.0000	-1.51	0.0000
TW11_3059	MFS transporter	-1.25	0.0000	-1.75	0.0000	-2.64	0.0000	-1.75	0.0000
TW11_3060	hydrolase	-0.51	0.1173	-0.81	0.0000	-1.25	0.0000	-1.11	0.0000
TW11_3061	ATP-binding protein	-0.04	0.9649	0.75	0.0002	1.09	0.0000	0.10	0.7911
TW11_3062	PadR family transcriptional regulator	1.03	0.0001	1.34	0.0000	1.00	0.0000	-0.03	0.9371
TW11_3064	threonine--tRNA ligase	-0.29	0.4554	-0.75	0.0000	-0.86	0.0000	-1.18	0.0000
TW11_3098	protein-glutamate O-methyltransferase CheR	-0.85	0.0055	-0.84	0.0000	-0.91	0.0000	-1.09	0.0000
TW11_3099	chemotaxis protein	-0.83	0.0152	-0.90	0.0000	-1.02	0.0000	-0.86	0.0011
TW11_3109	hypothetical protein	-0.71	0.0249	-1.19	0.0000	-1.14	0.0000	-1.04	0.0000
TW11_3110	membrane protein	-0.68	0.0150	-1.26	0.0000	-1.25	0.0000	-0.91	0.0001
TW11_3111	amidohydrolase	-0.50	0.1027	-1.07	0.0000	-1.10	0.0000	-0.95	0.0001
TW11_3131	galactitol-1-phosphate 5-dehydrogenase	-0.13	0.8830	0.16	0.6415	-0.10	0.7758	-1.09	0.0001
TW11_3132	phosphoribosylaminoimidazolesuccinocarboxamide	-0.20	0.6573	0.58	0.0003	1.43	0.0000	1.97	0.0000
TW11_3133	quaternary ammonium compound-resistance protein	-1.29	0.0003	-0.11	0.7253	0.15	0.5904	0.77	0.0090
TW11_3214	recombinase	0.12	0.9155	1.00	0.0028	0.42	0.2950	-0.58	0.2382
TW11_3241	dihydrodipicolinate reductase	-0.13	0.8832	0.18	0.5805	0.09	0.7868	-1.15	0.0004
TW11_3251	hypothetical protein	-0.37	0.5854	0.10	0.8156	-0.46	0.2053	-1.21	0.0004
TW11_3263	rRNA pseudouridine synthase	-0.63	0.0392	-0.83	0.0000	-1.18	0.0000	-0.90	0.0004
TW11_3265	fructose-1,6-bisphosphate aldolase, class II	-0.26	0.5487	-0.49	0.0044	-0.39	0.0628	-1.80	0.0000
TW11_3267	electron transfer flavoprotein subunit	0.04	0.9579	-0.91	0.0000	-0.97	0.0000	-1.13	0.0000
TW11_3268	electron transfer flavoprotein subunit beta/FixA	-0.06	0.9509	-1.31	0.0000	-1.32	0.0000	-1.16	0.0000
TW11_3269	acyl-CoA dehydrogenase	-0.04	0.9657	-1.40	0.0000	-1.32	0.0000	-1.13	0.0000
TW11_3270	2-hydroxyacyl-CoA dehydratase	-0.21	0.7437	-1.40	0.0000	-1.24	0.0000	-1.19	0.0000
TW11_3271	2-hydroxyglutaryl-CoA dehydratase	-0.17	0.8209	-1.48	0.0000	-1.35	0.0000	-1.25	0.0000
TW11_3272	2-hydroxyglutaryl-CoA dehydratase	-0.09	0.9302	-1.58	0.0000	-1.47	0.0000	-1.29	0.0000
TW11_3273	CoA transferase	-0.03	0.9742	-1.29	0.0000	-1.33	0.0000	-1.37	0.0000
TW11_3274	lactate dehydrogenase	0.05	0.9477	-0.42	0.1170	-0.50	0.0718	-1.55	0.0000
TW11_3275	TIGR04002 family protein	0.39	0.2945	-0.14	0.5371	-0.63	0.0015	-1.04	0.0000
TW11_3279	PRD domain-containing protein	0.50	0.2346	1.63	0.0000	2.37	0.0000	0.07	0.8359
TW11_3280	6-phospho-beta-glucosidase	0.53	0.2291	1.55	0.0000	2.31	0.0000	-0.03	0.9193

TW11_3281	PTS beta-glucoside transporter subunit EIIBCA	0.58	0.0873	**1.66**	**0.0000**	**2.34**	**0.0000**	-0.06	0.8594
TW11_3282	hypothetical protein	0.06	0.9477	0.06	0.8517	-0.10	0.7707	**-1.43**	**0.0000**
TW11_3290	ser/threonine protein phosphatase	-0.98	0.0002	-0.96	0.0000	**-1.06**	**0.0000**	**-1.05**	**0.0000**
TW11_3313	DedA family protein	**-1.72**	**0.0000**	-0.97	0.0005	-0.82	0.0030	-0.99	0.0038
TW11_3340	peptidase M20	-0.34	0.4380	-0.01	0.9696	-0.26	0.2393	**-1.20**	**0.0004**
TW11_3342	PTS fructose transporter subunit IID	-0.06	0.9458	-0.16	0.5371	-0.22	0.2996	**-1.08**	**0.0000**
TW11_3343	PTS sugar transporter subunit IIC	-0.14	0.8517	-0.40	0.1165	-0.47	0.0266	**-1.34**	**0.0000**
TW11_3351	pyridoxamine 5'-phosphate oxidase	**1.20**	**0.0000**	**1.25**	**0.0000**	**1.23**	**0.0000**	0.92	0.0003
TW11_3385	phosphoribosylformylglycinamidine synthase	-0.38	0.4214	-0.14	0.5391	0.56	0.0078	**1.93**	**0.0000**
TW11_3386	phosphoribosylamine--glycine ligase	-0.62	0.1628	-0.35	0.1531	0.50	0.0725	**2.09**	**0.0000**
TW11_3387	bifunctional	-0.62	0.1628	-0.22	0.3877	0.63	0.0096	**2.22**	**0.0000**
TW11_3388	phosphoribosylglycinamide formyltransferase	-0.47	0.2195	0.06	0.8286	0.73	0.0009	**2.18**	**0.0000**
TW11_3389	phosphoribosylformylglycinamidine cyclo-ligase	-0.46	0.2291	-0.01	0.9667	0.73	0.0008	**2.11**	**0.0000**
TW11_3390	amidophosphoribosyltransferase	-0.29	0.5542	0.08	0.7750	0.76	0.0010	**2.30**	**0.0000**
TW11_3391	phosphoribosylaminoimidazolesuccinocarboxamide	-0.22	0.6905	0.26	0.2829	0.82	0.0020	**2.22**	**0.0000**
TW11_3392	5-(carboxyamino)imidazole ribonucleotide mutase	0.13	0.8437	0.40	0.0928	**1.09**	**0.0000**	**2.32**	**0.0000**
TW11_3397	sugar-phosphate kinase	0.18	0.7840	0.10	0.7722	-0.05	0.8843	**-1.01**	**0.0006**
TW11_3402	GGDEF domain-containing protein	0.02	0.9774	0.82	0.0000	**1.43**	**0.0000**	0.93	0.0002
TW11_3409	transposase	-0.13	0.7823	-0.29	0.0794	-0.27	0.1334	**-1.06**	**0.0000**
TW11_3419	hypothetical protein	-0.42	0.4544	-0.80	0.0022	**-1.15**	**0.0000**	-0.32	0.4321
TW11_3425	orotate phosphoribosyltransferase	-0.67	0.0713	-0.21	0.3654	0.19	0.3484	**1.21**	**0.0000**
TW11_3426	dihydroorotate dehydrogenase	**-1.03**	**0.0001**	-0.37	0.0361	-0.06	0.8037	**1.07**	**0.0000**
TW11_3427	dihydroorotate dehydrogenase electron transfer	**-1.23**	**0.0000**	-0.81	0.0000	-0.34	0.1408	**1.12**	**0.0000**
TW11_3428	aspartate carbamoyltransferase	**-1.27**	**0.0000**	-0.72	0.0000	-0.31	0.1692	**1.06**	**0.0000**
TW11_3447	nitrogen regulation protein NR(I)	-0.72	0.0675	-0.82	0.0017	**-1.24**	**0.0000**	-0.93	0.0388
TW11_3459	membrane protein	0.86	0.0426	0.80	0.0000	0.74	0.0000	**1.03**	**0.0001**
TW11_3461	RNA-binding transcriptional accessory protein	-0.86	0.0527	**-1.37**	**0.0000**	**-1.54**	**0.0000**	**-1.13**	**0.0000**
TW11_3463	preprotein translocase subunit SecA	-0.34	0.5625	-0.83	0.0000	**-1.17**	**0.0000**	-0.95	0.0011
TW11_3488	acceptor oxidoreductase gamma subunit	-0.05	0.9479	-0.52	0.0016	-0.90	0.0000	**-1.04**	**0.0000**
TW11_3489	MFS transporter	-0.11	0.8548	-0.75	0.0000	**-1.18**	**0.0000**	**-1.17**	**0.0000**
TW11_3490	3-methyl-2-oxobutanoate dehydrogenase subunit	-0.15	0.8003	-0.85	0.0000	**-1.19**	**0.0000**	**-1.01**	**0.0000**
TW11_3491	2-oxoacid:acceptor oxidoreductase subunit delta	-0.20	0.6929	-0.87	0.0000	**-1.27**	**0.0000**	**-1.05**	**0.0000**
TW11_3492	ATP-binding protein	-0.14	0.8307	-0.92	0.0000	**-1.28**	**0.0000**	-0.87	0.0011
TW11_3493	butyrate kinase	0.06	0.9510	-0.87	0.0004	**-1.09**	**0.0000**	-0.68	0.0615
TW11_3516	anaerobic ribonucleoside-triphosphate reductase	**2.67**	**0.0000**	**3.70**	**0.0000**	**3.81**	**0.0000**	**3.02**	**0.0000**
TW11_3517	anaerobic ribonucleoside-triphosphate reductase	**2.78**	**0.0000**	**3.45**	**0.0000**	**3.50**	**0.0000**	**3.01**	**0.0000**
TW11_3552	50S ribosomal protein L17	-0.34	0.3781	-0.72	0.0000	-0.75	0.0000	**-1.04**	**0.0000**
TW11_3553	DNA-directed RNA polymerase subunit alpha	-0.68	0.0594	**-1.25**	**0.0000**	**-1.26**	**0.0000**	**-1.23**	**0.0000**

TW11_3554	30S ribosomal protein S4	-0.71	0.0737	**-1.27**	**0.0000**	**-1.30**	**0.0000**	**-1.16**	**0.0000**
TW11_3555	30S ribosomal protein S11	-0.68	0.1065	**-1.25**	**0.0000**	**-1.23**	**0.0000**	**-1.01**	**0.0001**
TW11_3556	30S ribosomal protein S13	-0.81	0.0369	**-1.36**	**0.0000**	**-1.33**	**0.0000**	**-1.06**	**0.0001**
TW11_3557	50S ribosomal protein L36	-0.84	0.0371	**-1.34**	**0.0000**	**-1.44**	**0.0000**	**-1.53**	**0.0000**
TW11_3558	translation initiation factor IF-1	-0.88	0.0147	**-1.29**	**0.0000**	**-1.39**	**0.0000**	**-1.37**	**0.0000**
TW11_3559	KOW domain-containing protein	-0.92	0.0103	**-1.46**	**0.0000**	**-1.46**	**0.0000**	**-1.35**	**0.0000**
TW11_3560	type I methionyl aminopeptidase	-0.92	0.0110	**-1.44**	**0.0000**	**-1.50**	**0.0000**	**-1.41**	**0.0000**
TW11_3561	adenylate kinase	-0.86	0.0293	**-1.45**	**0.0000**	**-1.55**	**0.0000**	**-1.44**	**0.0000**
TW11_3562	preprotein translocase subunit SecY	-0.85	0.0219	**-1.36**	**0.0000**	**-1.35**	**0.0000**	**-1.46**	**0.0000**
TW11_3563	50S ribosomal protein L15	-0.64	0.0864	**-1.05**	**0.0000**	-0.91	0.0000	**-1.02**	**0.0001**
TW11_3564	50S ribosomal protein L30	-0.75	0.0504	**-1.24**	**0.0000**	**-1.19**	**0.0000**	**-1.11**	**0.0000**
TW11_3565	30S ribosomal protein S5	-0.71	0.1342	**-1.40**	**0.0000**	**-1.35**	**0.0000**	**-1.05**	**0.0006**
TW11_3566	50S ribosomal protein L18	-0.84	0.0497	**-1.53**	**0.0000**	**-1.51**	**0.0000**	**-1.17**	**0.0001**
TW11_3567	50S ribosomal protein L6	-0.91	0.0247	**-1.50**	**0.0000**	**-1.50**	**0.0000**	**-1.35**	**0.0000**
TW11_3568	30S ribosomal protein S8	-0.88	0.0361	**-1.54**	**0.0000**	**-1.44**	**0.0000**	**-1.20**	**0.0001**
TW11_3569	30S ribosomal protein S14 type Z	-0.78	0.0543	**-1.35**	**0.0000**	**-1.33**	**0.0000**	**-1.03**	**0.0003**
TW11_3570	50S ribosomal protein L5	-0.98	0.0144	**-1.57**	**0.0000**	**-1.54**	**0.0000**	**-1.35**	**0.0000**
TW11_3571	50S ribosomal protein L24	-0.83	0.0565	**-1.45**	**0.0000**	**-1.43**	**0.0000**	**-1.07**	**0.0004**
TW11_3572	50S ribosomal protein L14	-0.99	0.0125	**-1.55**	**0.0000**	**-1.47**	**0.0000**	**-1.16**	**0.0001**
TW11_3573	30S ribosomal protein S17	-0.82	0.0479	**-1.34**	**0.0000**	**-1.31**	**0.0000**	**-1.07**	**0.0003**
TW11_3574	50S ribosomal protein L29	**-1.02**	**0.0073**	**-1.54**	**0.0000**	**-1.46**	**0.0000**	**-1.28**	**0.0000**
TW11_3575	50S ribosomal protein L16	-0.87	0.0396	**-1.41**	**0.0000**	**-1.29**	**0.0000**	**-1.07**	**0.0003**
TW11_3576	30S ribosomal protein S3	-1.00	0.0175	**-1.60**	**0.0000**	**-1.51**	**0.0000**	**-1.21**	**0.0000**
TW11_3577	50S ribosomal protein L22	**-1.01**	**0.0096**	**-1.56**	**0.0000**	**-1.47**	**0.0000**	**-1.26**	**0.0000**
TW11_3578	30S ribosomal protein S19	-0.96	0.0124	**-1.47**	**0.0000**	**-1.43**	**0.0000**	**-1.27**	**0.0000**
TW11_3579	50S ribosomal protein L2	-0.94	0.0117	**-1.49**	**0.0000**	**-1.43**	**0.0000**	**-1.21**	**0.0000**
TW11_3580	50S ribosomal protein L23	-0.90	0.0153	**-1.46**	**0.0000**	**-1.42**	**0.0000**	**-1.12**	**0.0001**
TW11_3581	50S ribosomal protein L4	-0.94	0.0157	**-1.54**	**0.0000**	**-1.51**	**0.0000**	**-1.19**	**0.0000**
TW11_3582	50S ribosomal protein L3	-0.97	0.0094	**-1.67**	**0.0000**	**-1.66**	**0.0000**	**-1.33**	**0.0000**
TW11_3583	30S ribosomal protein S10	-0.93	0.0022	**-1.63**	**0.0000**	**-1.63**	**0.0000**	**-1.28**	**0.0000**
TW11_3584	elongation factor Tu	-0.46	0.3472	**-1.16**	**0.0000**	**-1.05**	**0.0000**	-0.72	0.0083
TW11_3585	elongation factor G	-0.67	0.0959	**-1.27**	**0.0000**	**-1.11**	**0.0000**	-0.93	0.0017
TW11_3586	30S ribosomal protein S7	-0.69	0.0817	**-1.37**	**0.0000**	**-1.23**	**0.0000**	-0.86	0.0024
TW11_3587	30S ribosomal protein S12	-0.74	0.0227	**-1.31**	**0.0000**	**-1.13**	**0.0000**	-0.87	0.0008
TW11_3595	transcription termination/antitermination factor	-0.55	0.1195	**-1.10**	**0.0000**	-0.97	0.0000	-0.77	0.0019
TW11_3596	preprotein translocase subunit SecE	-0.55	0.0945	**-1.12**	**0.0000**	-0.96	0.0000	-0.68	0.0061
TW11_3598	elongation factor Tu	-0.36	0.5025	**-1.10**	**0.0000**	**-1.08**	**0.0000**	-0.76	0.0063
TW11_3599	RNA polymerase sporulation sigma factor SigH	-0.46	0.4888	**-1.29**	**0.0000**	**-1.42**	**0.0000**	**-1.20**	**0.0001**

TW11_3600	NYN domain-containing protein	-0.49	0.3901	**-1.20**	**0.0000**	**-1.38**	**0.0000**	**-1.34**	**0.0000**
TW11_3601	23S rRNA	-0.45	0.4368	**-1.08**	**0.0000**	**-1.32**	**0.0000**	**-1.24**	**0.0000**
TW11_3602	thymidylate synthase (FAD)	-0.40	0.4576	**-1.08**	**0.0000**	**-1.36**	**0.0000**	**-1.31**	**0.0000**
TW11_3608	proline--tRNA ligase	-0.46	0.2208	**-1.12**	**0.0000**	**-1.13**	**0.0000**	-0.63	0.0120
TW11_3614	PTS system sugar-specific permease component	-0.33	0.4919	-0.31	0.2396	-0.66	0.0059	**-1.16**	**0.0001**
TW11_3618	dihydrolipoyl dehydrogenase	-0.11	0.8315	-0.30	0.1198	-0.65	0.0006	**-1.19**	**0.0000**
TW11_3620	alpha-ketoacid dehydrogenase subunit beta	-0.12	0.8342	-0.47	0.0349	-0.49	0.0236	**-1.21**	**0.0000**
TW11_3635	protein arginine kinase	0.06	0.9510	-0.96	0.0001	**-1.13**	**0.0000**	-0.22	0.6096
TW11_3689	hypothetical protein	0.87	0.0098	**1.16**	**0.0000**	**1.02**	**0.0000**	**1.83**	**0.0000**
TW11_3697	mechanosensitive ion channel protein	**-1.24**	**0.0000**	-0.86	0.0000	-0.94	0.0000	-0.88	0.0009
TW11_3698	transcription repressor NadR	**-1.30**	**0.0000**	-0.94	0.0000	-0.94	0.0000	-0.80	0.0039
TW11_3705	hypothetical protein	0.96	0.0032	**1.18**	**0.0000**	**1.10**	**0.0000**	**1.96**	**0.0000**
TW11_3713	mechanosensitive ion channel protein	**-1.29**	**0.0000**	-0.97	0.0000	**-1.00**	**0.0000**	-0.96	0.0003
TW11_3714	transcription repressor NadR	**-1.15**	**0.0002**	-0.86	0.0000	-0.93	0.0000	-0.73	0.0079
TW11_3730	DNA topoisomerase (ATP-hydrolyzing) subunit B	-0.47	0.4390	**-1.10**	**0.0000**	**-1.01**	**0.0000**	-0.38	0.2606
TW11_3755	hypothetical protein	0.29	0.4730	0.47	0.0220	0.59	0.0441	**-1.04**	**0.0007**
TW11_3757	replication protein	-0.28	0.5569	0.55	0.0021	**1.17**	**0.0000**	0.39	0.2869
TW11_3765	hypothetical protein	**-1.30**	**0.0067**	**-1.06**	**0.0043**	-0.85	0.0219	-0.35	0.5449
TW11_3769	hypothetical protein	0.35	0.4285	0.14	0.5576	0.68	0.0013	**1.46**	**0.0000**
TW11_3770	hypothetical protein	0.29	0.5569	-0.08	0.7527	0.54	0.0151	**1.19**	**0.0000**
TW11_3771	hypothetical protein	0.38	0.6323	0.27	0.5629	0.96	0.0077	**1.52**	**0.0003**
TW11_3772	recombinase family protein	0.25	0.5656	0.11	0.6026	0.62	0.0006	**1.95**	**0.0000**
TW11_3773	mannosyl-glycoprotein	0.47	0.1497	0.52	0.0013	0.61	0.0003	**1.25**	**0.0000**
TW11_3777	bacterial regulatory s, luxR family protein	0.11	0.8658	0.75	0.0000	**2.25**	**0.0000**	**4.20**	**0.0000**
TW11_3778	hypothetical protein	0.39	0.3406	0.74	0.0000	**1.84**	**0.0000**	**3.53**	**0.0000**

Genes was considered DE if they RPKM⩾5 and |log2(FC)|⩾1 and FDR⩽0.01. Positive and negative values indicate an increase and decrease in transcript levels during infection with significant differences shown in red and blue, respectively.
