# Supplementary material for: Isolation and Characterization of the Novel Phage JD032 and Global Transcriptomic Response during JD032 Infection of Clostridioides difficile Ribotype 078
Source: mSystems. 2020 May 5;5(3):e00017-20. doi: 10.1128/mSystems.00017-20 (PMC7205517; doi:10.1128/mSystems.00017-20)
Supplement: TABLE S7 [file mSystems.00017-20-st007.pdf]

Table S7. Significantly enriched KEGG pathways of DEGs in *C. difficile* strain TW11

Significantly regulated pathways[a]	Number of DEGs[b]			
	30min p.i	45min p.i	75min p.i	135min p.i
**Carbohydrate metabolism**				
00010 Glycolysis / Gluconeogenesis [PATH:ko00010]		2	2	14
00020 Citrate cycle (TCA cycle) [PATH:ko00020]			2	4
00030 Pentose phosphate pathway [PATH:ko00030]				5
00040 Pentose and glucuronate interconversions [PATH:ko00040]	2	1		2
00051 Fructose and mannose metabolism [PATH:ko00051]	4	1	2	12
00052 Galactose metabolism [PATH:ko00052]	5	5		8
00500 Starch and sucrose metabolism [PATH:ko00500]	3	6	5	
00520 Amino sugar and nucleotide sugar metabolism [PATH:ko00520]	1	2	1	7
00630 Glyoxylate and dicarboxylate metabolism [PATH:ko00630]	1	2	4	2
00620 Pyruvate metabolism [PATH:ko00620]		1	1	8
00640 Propanoate metabolism [PATH:ko00640]				2
00650 Butanoate metabolism [PATH:ko00650]				5
00562 Inositol phosphate metabolism [PATH:ko00562]				1
**Membrane transport**				
02010 ABC transporters [PATH:ko02010]			4	
02060 Phosphotransferase system (PTS) [PATH:ko02060]	8	7	5	
**Nucleotide metabolism**				
00230 Purine metabolism [PATH:ko00230]				9
00240 Pyrimidine metabolism [PATH:ko00240]				8
**Translation**				
03010 Ribosome [PATH:ko03010]		27	26	26
00970 Aminoacyl-tRNA biosynthesis [PATH:ko00970]		1	4	2
**Energy metabolism**				
00190 Oxidative phosphorylation [PATH:ko00190]			6	
00195 Photosynthesis [PATH:ko00195]			6	
00710 Carbon fixation in photosynthetic organisms [PATH:ko00710]			6	
00680 Methane metabolism [PATH:ko00680]			3	
00910 Nitrogen metabolism [PATH:ko00910]			2	

[a]Differentially expressed host genes for each condition were subjected to gene set enrichment analysis (GSEA) on annotated Kyoto Encyclopedia of Genes and Genomes (KEGG) pathways using GAGE with an FDR cutoff of <0.05.

[b]Up-regulated genes and down-regulated genes shown in red and green, respectively.
