# Supplementary material for: Isolation and Characterization of the Novel Phage JD032 and Global Transcriptomic Response during JD032 Infection of Clostridioides difficile Ribotype 078
Source: mSystems. 2020 May 5;5(3):e00017-20. doi: 10.1128/mSystems.00017-20 (PMC7205517; doi:10.1128/mSystems.00017-20)
Supplement: TEXT S1 [file mSystems.00017-20-s0001.docx]

**The viability of *C. difficile* TW11 at 135 minutes after phage JD032 infection**

In the stable phase (135min), the phage JD032 and the host TW11 are in a relatively balanced state, wherein most TW11 cells were lysed. Therefore, the extracted RNA at 135min included a mixed population of viable and dead *C. difficile* cells. However, the currently used technology cannot precisely determine the ratio of viable bacteria to dead bacteria, thus, we speculated a ratio based on the existing theoretical and experimental data as follows:

As the entire infection process is completed during the logarithmic growth phase of the bacteria, therefore, we can estimate a corresponding ratio based on the relationship between the OD600 values and the amount of viable bacteria tested during the logarithmic growth phase (Fig. S1).

When infected with phage JD032 for 1 h, 2 h and 3 h, the OD600 values of the TW11 culture were 0.618, 0.147, and 0.114, respectively (Fig.3). According to the calculation formula obtained in Fig. S1, it can be estimated that the number of corresponding viable bacteria were 4.6×10^7^ CFU/mL, 6.7×10^6^ CFU/mL and 5.9×10^6^ CFU/mL, respectively. So, at 135 min, the number of viable bacteria should be between 5.9×10^6^ CFU /mL-6.7×10^6^ CFU/mL. Suppose that the bacteria no longer multiply between 1 h and 3 h after phage infection. Then, the rough number of total bacteria was 4.6×10^7^ CFU/mL, and the dead bacteria was between 3.93×10^7^CFU/mL- 4.01×10^7^CFU/mL. The corresponding ratio of viable to dead bacteria was between 17.05% -14.71%. However, during phage infection, a small number of uninfected bacteria may continue to multiply, so the number of total bacteria was more than 6×10^7^ CFU/mL. Therefore, the ratio of viable / dead bacteria should be less than 17.05%.
