# Supplementary figures and images for: Isolation and Characterization of the Novel Phage JD032 and Global Transcriptomic Response during JD032 Infection of Clostridioides difficile Ribotype 078
Source: mSystems. 2020 May 5;5(3):e00017-20. doi: 10.1128/mSystems.00017-20 (PMC7205517; doi:10.1128/mSystems.00017-20)

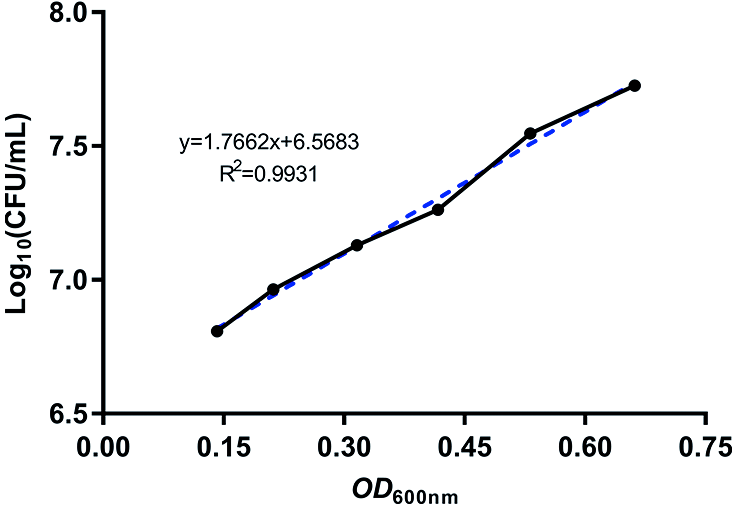

Supplement: FIG S1 [file mSystems.00017-20-sf001.tif]
