# Supplementary material for: Isolation and Characterization of the Novel Phage JD032 and Global Transcriptomic Response during JD032 Infection of Clostridioides difficile Ribotype 078
Source: mSystems. 2020 May 5;5(3):e00017-20. doi: 10.1128/mSystems.00017-20 (PMC7205517; doi:10.1128/mSystems.00017-20)
Supplement: TABLE S8 [file mSystems.00017-20-st008.pdf]

Table S8. Primers used in this study

Primer	Sequence (5'->3')	Product length(bp)
TW11_1325F	TGGTACTACTGGAAAAGGAATGAG	129
TW11_1325R	ATTGCCTTTACAAATCCATCTGC	
TW11_3516F	AGTGCCATAATCCTCAAACGCA	123
TW11_3516R	ATGGTTCTCCTCCTGATAGCGT	
TW11_2708F	GCATTAGCTGGAAGTGTGTTGT	121
TW11_2708R	TTCCCAAAGATGTTGCTGGAT	
TW11_1443F	CAGGTGATTCAATGGTAGCAGG	101
TW11_1443R	GGCAGTTGCGCTACCTGAAG	
TW11_0859F	CAGAGATTACTATCTGACGAGTGG	174
TW11_0859R	TCGCTATATCTTCCAGGCTGTAT	
TW11_0231F	AGTTCAGATGGAGAACAGTAACAGA	123
TW11_0231R	TGACCCATTTCTCTATAACCCGT	
TW11_0232F	AGGGAGGCGTTAGACCTGTT	145
TW11_0232R	AGCCCATATTCATTGGAACTTATCTC	
TW11_1632F	TGAAATACTTCCTAAGTTGTCATTAAGT	191
TW11_1632R	TCTTCATCTGTATTTGCATACTCTCA	
Porf014F	CGTAGTTGCGTTTGGAGAAGT	162
Porf014R	TGCTTTCGTTCTTCATCTCCGT	
Porf016F	AGCCCTCAAAGCAAACGCAT	167
Porf016R	ACACTCCAAGCGTCACAAATG	
Porf021F	GCTATCACCTTTACCAGCCAGA	120
Porf021R	GCTGTCAAATCGTAGCGTGG	
Porf025F	GGGCTTGTAAGTGTGATTGTGG	83
Porf025R	GCATCCACATGATTTAGTTCTACCA	
gluD-F	ATGCAGTAGGGCCAACAAAA	135
gluD-R	TTCCACCTTTACCTCCACCA	
